# Na_V_1.6 dysregulation within myocardial T-tubules by D96V calmodulin enhances proarrhythmic sodium and calcium mishandling

**DOI:** 10.1172/JCI152071

**Published:** 2023-04-03

**Authors:** Mikhail Tarasov, Heather L. Struckman, Yusuf Olgar, Alec Miller, Mustafa Demirtas, Vladimir Bogdanov, Radmila Terentyeva, Andrew M. Soltisz, Xiaolei Meng, Dennison Min, Galina Sakuta, Izabella Dunlap, Antonia D. Duran, Mark P. Foster, Jonathan P. Davis, Dmitry Terentyev, Sándor Györke, Rengasayee Veeraraghavan, Przemysław B. Radwański

**Affiliations:** 1 The Frick Center for Heart Failure and Arrhythmia, Dorothy M. Davis Heart and Lung Research Institute, College of Medicine, The Ohio State University Wexner Medical Center, Columbus, Ohio, USA.; 2 Division of Outcomes and Translational Sciences, College of Pharmacy,; 3 Department of Biomedical Engineering, College of Engineering,; 4 Department of Physiology and Cell Biology, College of Medicine,; 5 Department of Chemistry and Biochemistry, The Ohio State University, Columbus, Ohio, USA.

**Keywords:** Cardiology, Cell Biology, Arrhythmias, Calmodulin, Sodium channels

## Abstract

Calmodulin (CaM) plays critical roles in cardiomyocytes, regulating Na^+^ (Na_V_) and L-type Ca^2+^ channels (LTCCs). LTCC dysregulation by mutant CaMs has been implicated in action potential duration (APD) prolongation and arrhythmogenic long QT (LQT) syndrome. Intriguingly, D96V-CaM prolongs APD more than other LQT-associated CaMs despite inducing comparable levels of LTCC dysfunction, suggesting dysregulation of other depolarizing channels. Here, we provide evidence implicating Na_V_ dysregulation within transverse (T) tubules in D96V-CaM–associated arrhythmias. D96V-CaM induced a proarrhythmic late Na^+^ current (I_Na_) by impairing inactivation of Na_V_1.6, but not the predominant cardiac Na_V_ isoform Na_V_1.5. We investigated arrhythmia mechanisms using mice with cardiac-specific expression of D96V-CaM (cD96V). Super-resolution microscopy revealed close proximity of Na_V_1.6 and RyR2 within T-tubules. Na_V_1.6 density within these regions increased in cD96V relative to WT mice. Consistent with Na_V_1.6 dysregulation by D96V-CaM in these regions, we observed increased late Na_V_ activity in T-tubules. The resulting late I_Na_ promoted aberrant Ca^2+^ release and prolonged APD in myocytes, leading to LQT and ventricular tachycardia in vivo. Cardiac-specific Na_V_1.6 KO protected cD96V mice from increased T-tubular late Na_V_ activity and its arrhythmogenic consequences. In summary, we demonstrate that D96V-CaM promoted arrhythmias by dysregulating LTCCs and Na_V_1.6 within T-tubules and thereby facilitating aberrant Ca^2+^ release.

## Graphical abstract

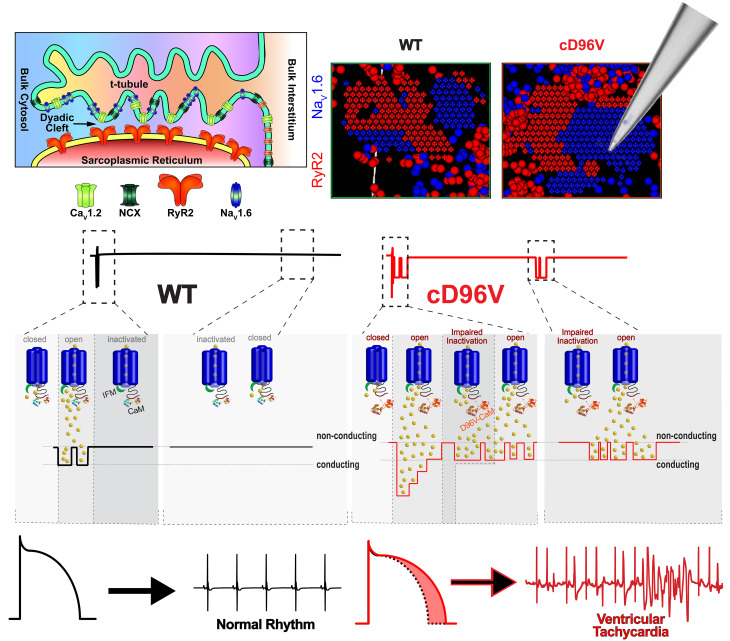

## Introduction

Calmodulin (CaM) regulates many different ion channels across multiple organs, including the heart. Mutations in CaM have been linked to Ca^2+^ mishandling and arrhythmias, manifesting as long QT (LQT) syndrome, catecholaminergic polymorphic ventricular tachycardia (VT), or idiopathic ventricular fibrillation (1). CaM-associated arrhythmia syndromes, termed calmodulinopathies, have been linked with dysregulation of Ca_v_1.2, RyR2, K_v_7.1, and SK channels (2–7); however, to date, only direct dysregulation of the Ca^2+^-handling machinery has been linked to arrhythmias in vivo (8). In particular, LQT-associated CaM mutations impair inactivation of the Ca^2+^ current (I_Ca_), prolonging action potential duration (APD) and inducing LQT (7). Intriguingly, however, the CaM mutant associated with the most severe APD prolongation in animal models, D96V-CaM, does not induce commensurate enhancement of the I_Ca_ (7). This has led to the hypothesis that impaired inactivation of other inward currents, such as the sodium current (I_Na_), may also contribute to calmodulinopathies. In support of this hypothesis, and despite the fact that the D96V-CaM mutation did not elicit the longest QT interval in a clinical setting (9), Na^+^ channel (Na_V_) blockade with mexiletine reduced the heart rate–corrected QT (QTc) interval in a patient harboring the D96V-CaM mutation (3). Adding impetus to this idea is the identification of Na_V_ carboxy terminal domains (CTDs), the site of CaM interaction, as a hotspot for mutations associated with electrophysiological dysfunction in the heart and the brain. Specifically, Na_V_CTD mutations that disrupt CaM interaction impair inactivation and induce pathogenic “late” or “persistent” I_Na_ (10–12). This suggests that LQT syndrome, resulting from defects in Na_V_CTD and calmodulinopathies, may represent 2 sides of the same mechanistic coin. However, early attempts to implicate Na_V_s in calmodulinopathies, which focused on the predominant cardiac isoform Na_V_1.5, proved inconclusive (13, 14).

Some clues to help resolve this conundrum may be gleaned from studies in the brain, which linked CTD defects in Na_V_1.2 and Na_V_1.6 with impaired inactivation and consequent epilepsy (10, 11). Therefore, we hypothesized that the key to understanding cardiac calmodulinopathies may lie with less abundant, tetrodotoxin-sensitive (TTX-sensitive) Na_V_s, such as Na_V_1.6. Indeed, multiple neuronal-type Na_V_s, including Na_V_1.6, have been identified in the heart (15–21) and implicated in arrhythmogenic dysregulation of Na^+^-Ca^2+^ cycling in multiple disease states (22–27). Intriguingly, the Na_V_ isoform with the lowest CaM affinity, Na_V_1.6 (10, 11), exhibits the largest magnitude of late I_Na_ relative to peak I_Na_, suggesting it may be a candidate for investigation vis-à-vis calmodulinopathies.

In this study, we reveal what we believe to be previously unrecognized CaM-mediated dysregulation of Na_V_s, which contributes to calmodulinopathy. We report an arrhythmogenic concept, whereby impaired Na_V_1.6 inactivation in the presence of D96V-CaM contributed to abnormal Na^+^/Ca^2+^ handling within T-tubule nanodomains. In turn, this led to aberrant Ca^2+^ release and APD prolongation on the cellular level and in arrhythmias in vivo.

## Results

### D96V-CaM promotes cellular arrhythmia precursors through a subset of TTX-sensitive Na_V_s.

**Figure 1:**
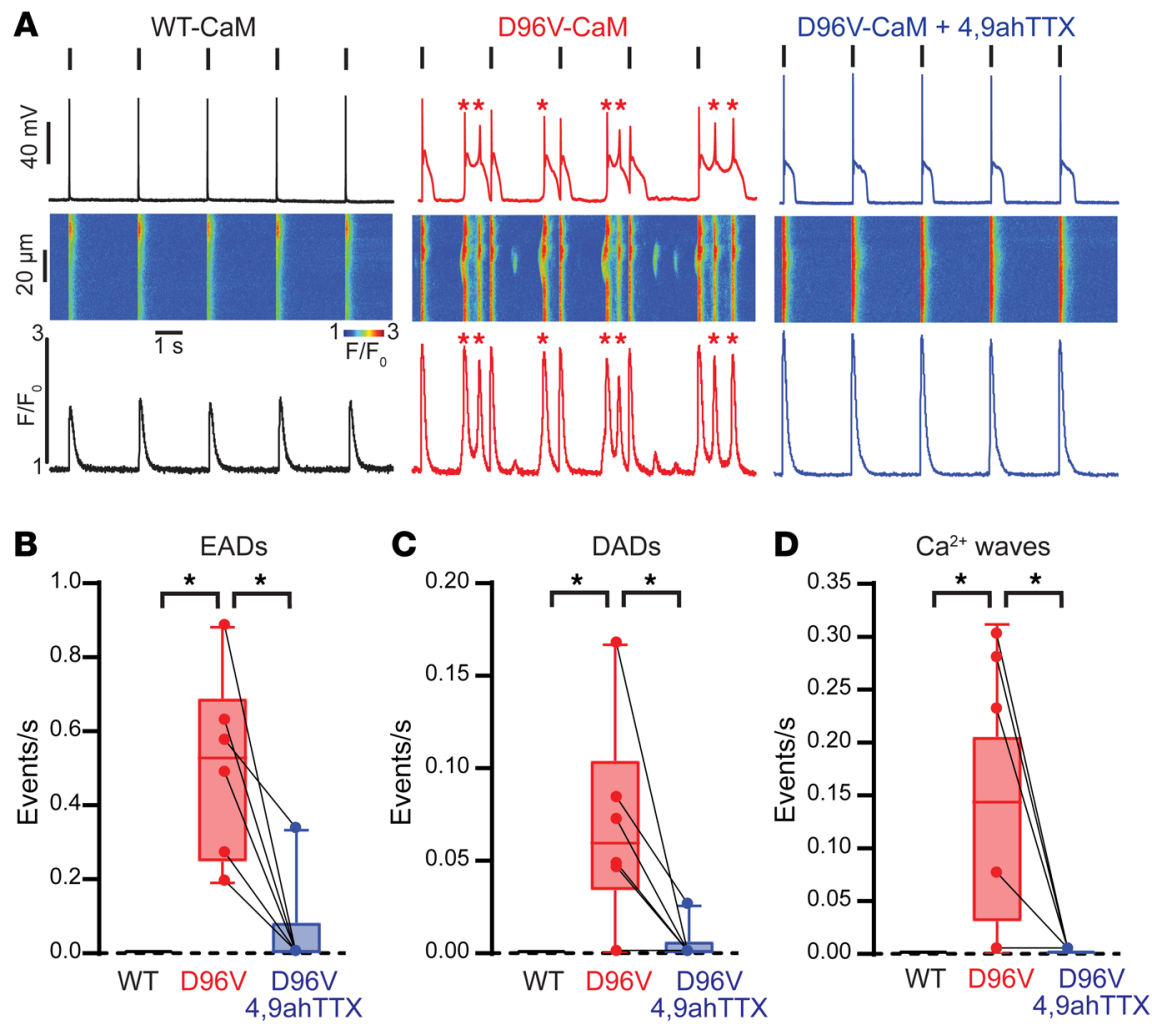
D96V-CaM promotes Na_V_-mediated cellular arrhythmias. (**A**) Simultaneous AP (top) and Ca^2+^ imaging (linescan, middle and corresponding averaged intensity Ca^2+^ transient, bottom) from isolated WT murine cardiomyocytes dialyzed with 6.5 μM WT CaM (left panel) and D96V-CaM in the absence (middle panel) and presence (right panel) of 300 nM 4,9ahTTX. Black vertical lines above the AP recordings mark the electrical stimuli. Red asterisks indicate EADs and DADs and the corresponding Ca^2+^ waves. In these experiments, the recombinant CaMs were not FLAG tagged. (**B**) EADs, (**C**) DADs, and (**D**) Ca^2+^ wave frequencies. For D96V-CaM and D96V-CaM plus 4,9ahTTX, *n* = 6 cells from 4 mice (*n* = 3 males, *n* = 1 female, 14–17 weeks old), and for WT CaM, *n* = 3 cells from 3 male mice (7–10 weeks old). Values from the same cells dialyzed with D96V-CaM in the absence and presence of 4,9ahTTX are connected with lines. *q < 0.05, by Kruskal-Wallis test with the original FDR method of Benjamini and Hochberg for multiple comparisons.

We used WT murine cardiomyocytes to examine the effect of the LQT-associated mutant CaM D96V-CaM on cellular arrhythmia potential using simultaneous patch-clamp recordings of action potentials (APs) and confocal imaging of Ca^2+^ release. We found that D96V-CaM (6.5 μM), but not WT CaM (6.5 μM), when introduced through the patch pipette (dialysis), significantly increased the frequency of Ca^2+^ waves (Figure 1, A and D). This, in turn, resulted in early afterdepolarizations (EADs) and delayed afterdepolarizations (DADs) (Figure 1, A–C), consistent with the LQT cellular arrhythmia phenotype of D96V-CaM (28).

When compared with other LQT-associated mutant CaMs, D96V-CaM promotes a similar degree of dysfunction in L-type Ca^2+^ channels (LTCCs) (7), but comparatively much more profound APD prolongation. This suggests that D96V-CaM probably dysregulates other effector targets beyond Ca_V_1.2 that likely include depolarizing channels, given the extent of APD prolongation and cellular arrhythmia potential. Thus, we investigated the contribution of I_Na_ dysfunction to calmodulinopathy. In this context, our previous work highlights the TTX-sensitive Na_V_1.6 isoform as a particularly relevant target (21, 29, 30). We therefore conducted preliminary proof-of-principle studies using a 300 nM dose of 4,9-anhydrotetrodotoxin (4,9ahTTX) (31), a concentration, which achieves greater than 80% blockage of murine and human Na_V_1.6 without affecting Na_V_1.5 (Supplemental Figure 10; supplemental material available online with this article; https://doi.org/10.1172/JCI152071DS1). 4,9ahTTX mitigated D96V-CaM–induced Ca^2+^ mishandling (Ca^2+^ waves; Figure 1, A and D) and cellular arrhythmias (EADs and DADs; Figure 1, A–C) and shortened the APD (Supplemental Figure 11). Notably, the APD in the presence of D96V-CaM and 4,9ahTTX was still prolonged relative to baseline levels, as evidenced by WT CaM dialysis, delineating the respective contributions of LTCC and Na_V_1.6 dysregulation to LQT and arrhythmias in calmodulinopathy.

### D96V-CaM impairs inactivation of TTX-sensitive I_Na_ in murine and human iPSC-derived cardiomyocytes.

**Figure 2:**
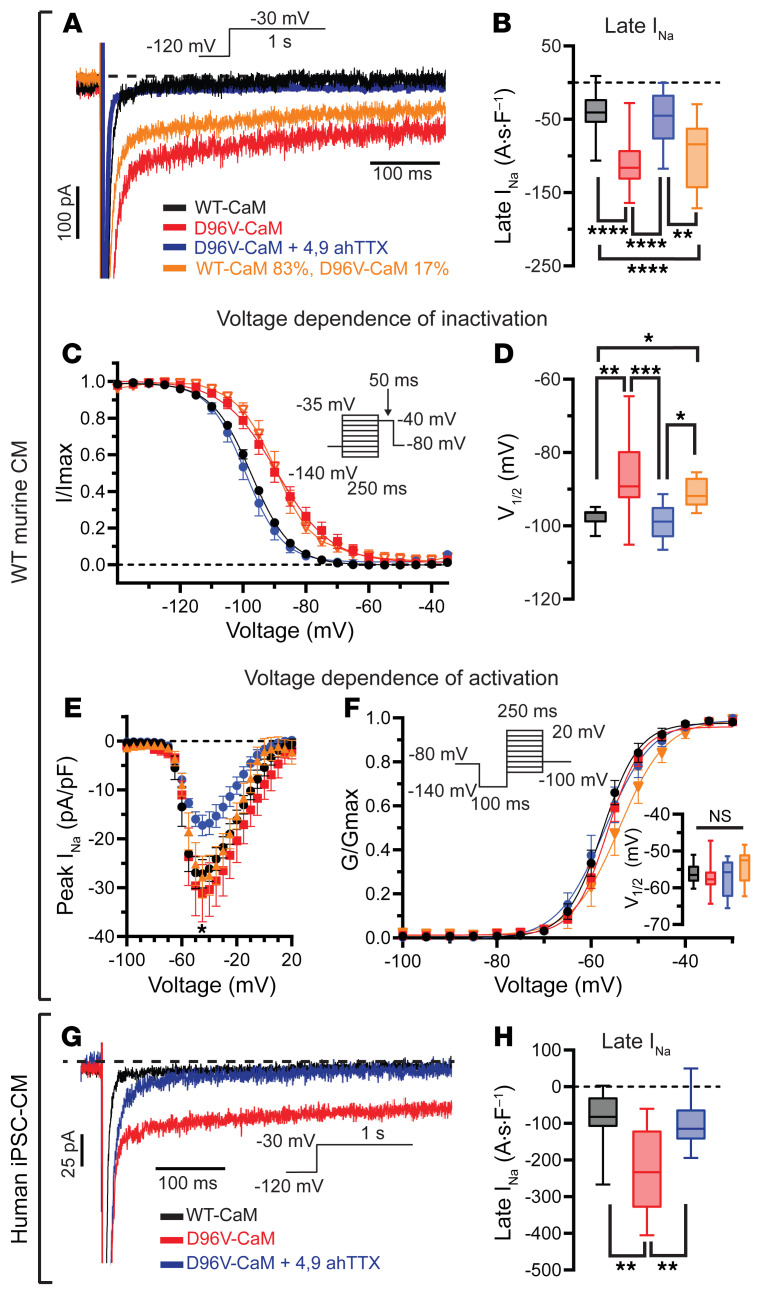
D96V-CaM impairs I_Na_ inactivation in murine and human iPSC-CMs. (**A**) Representative late I_Na_ traces recorded in WT murine cardiomyocytes dialyzed with 6.5 μM WT CaM (black), 6.5 μM D96V-CaM in the absence (red) or presence (blue) of 300 nM of 4,9ahTTX, and 5.4 μM WT CaM (83%) plus 1.1 μM D96V-CaM (17%; orange). The voltage protocol is illustrated above the traces. In these experiments, the recombinant CaMs were not FLAG tagged. (**B**) Summary: Late I_Na_ integral. For WT CaM, *n* = 21 cells from 9 mice (*n* = 4 females, *n* = 5 males, 6–26 weeks old); D96V-CaM *n* = 23 cells from 11 mice (*n* = 6 males, *n* = 5 females, 6–15 weeks old); D96V-CaM plus 4.9ahTTX, *n* = 9 cells from 6 mice (*n* = 3 males, *n* = 3 females, 6–15 weeks old); 5.4 μM WT CaM (83%) plus 1.1 μM D96V-CaM (17%), *n* = 17 cells from 8 mice (*n* = 4 males, *n* = 4 females, 12–24 weeks old). **q < 0.01 and ****q < 0.0001, by ordinary 1-way ANOVA test with the original FDR method of Benjamini and Hochberg for multiple comparisons. (**C**) Steady-state inactivation curves and (**D**) the corresponding half-maximal voltage (V_1/2_) of inactivation. For WT CaM, *n* = 12 cells from 6 mice (*n* = 2 females, *n* = 4 males, 6–26 weeks old); D96V-CaM, *n* = 19 cells from 8 mice (*n* = 5 males, *n* = 3 females, 6–25 weeks old); D96V-CaM plus 4.9ahTTX (300 nM), *n* = 10 cells from 5 mice (*n* = 2 males, *n* = 3 females, 6–25 weeks old), 5.4 μM WT CaM (83%) plus 1.1 μM D96V-CaM (17%), *n* = 10 cells from 7 mice (*n* = 4 males, *n* = 3 females, 11–24 weeks old). *q < 0.05, **q < 0.01, and ***q < 0.001, by ordinary 1-way ANOVA with the original FDR method of Benjamini and Hochberg for multiple comparisons. (**E**) Peak I_Na_ I–V relationship and (**F**) normalized I_Na_ conductance with the corresponding V_1/2_ of activation (inset). For WT CaM, *n* = 13 cells from 7 mice (*n* = 4 males, *n* = 3 females, 6–26 weeks old); D96V-CaM *n* = 10, *n* = 7 (*n* = 5 males, *n* = 2 females, 6–25 weeks old); D96V-CaM plus 4.9ahTTX (300 nM) *n* = 9, *n* = 7 (*n* = 4 males, *n* = 3 females, 6–25 weeks old); 5.4 μM WT CaM (83%) plus 1.1 μM D96V-CaM (17%) *n* = 6, *n* = 3 (*n* = 2 males, *n* = 1 female, 12–18 weeks old). *q < 0.05 for peak I_Na_ of D96V-CaM plus 4.9ahTTX versus D96V-CaM at –45 mV. *q* > 0.05 (NS), by Kruskal-Wallis test with the original FDR method of Benjamini and Hochberg for multiple comparisons. (**G**) Representative late I_Na_ recorded from human iPSC-CMs dialyzed with 6.5 μM WT CaM (black) or D96V-CaM in the absence (red) or presence (blue) of 300 nM 4,9ahTTX. (**H**) Summary: Late I_Na_ integral. For WT CaM, *n* = 14; D96V-CaM, *n* = 17; D96V-CaM plus 4.9ahTTX (300 nM), *n* = 11. **q < 0.01, by Kruskal-Wallis test with the original FDR method of Benjamini and Hochberg for multiple comparisons. A∙s∙F^–1^, amperes seconds per Farad; G/Gmax, normalized membrane conductance.

Next, we directly examined dysregulation of I_Na_ by D96V-CaM in WT murine cardiomyocytes. Unlike the heterologous expression systems previously used to study mutant CaM effects on a single Na_V_ isoform (13, 14), cardiomyocytes allow the examination of multiple Na_V_ isoforms that are expressed in their native environment and which together comprise I_Na_. Dialysis of D96V-CaM (6.5 μM) produced a significant increase in late I_Na_ relative to WT CaM (6.5 μM) (Figure 2, A and B). This is consistent with the marked APD prolongation we observed (Supplemental Figure 11) and is indicative of impaired I_Na_ inactivation. Indeed, voltage dependence of I_Na_ inactivation exhibited a depolarizing shift with D96V-CaM compared with WT CaM (Figure 2, C and D). In line with the notion that Na_V_1.6 is likely the Na_V_ isoform affected by D96V-CaM, changes induced by this LQT-associated CaM mutant in I_Na_ inactivation were abrogated by 300 nM 4,9ahTTX (Figure 2, A–D). Of note, peak I_Na_ in WT myocytes was not affected by D96V-CaM (Figure 2, E and F), however, Na_V_1.6 inhibition (300 nM 4,9ahTTX) reduced peak I_Na_ by 46.92% ± 1.66% under these conditions (Figure 2E), consistent with previous findings (21, 29).

Since, CaM is encoded by 3 *CALM* genes (thus, 6 alleles), a dominant-negative mutation in one of these alleles is expected to produce a mixture of approximately 17% mutant CaM and approximately 83% WT CaM. To verify the aforementioned proarrhythmic effects with this physiologic mutant CaM/WT CaM ratio, we dialyzed WT murine cardiomyocytes with 5.4 μM WT CaM plus 1.1 μM D96V-CaM. Despite the reduced D96V-CaM concentration, we observed a similar degree of I_Na_ dysfunction, as with a higher D96V-CaM concentration (Figure 2, A–F). These results highlight the potential pathophysiological impact of dominant-negative D96V-CaM on I_Na_ dysfunction in calmodulinopathy.

Furthermore, to examine the translatability of our findings with D96V-CaM in murine cardiomyocytes to human physiology, we used human induced pluripotent stem cell–derived ventricular cardiomyocytes (iPSC-CMs). First, we confirmed the expression of Na_V_1.6 in human iPSC-CMs on both mRNA and protein levels (Supplemental Figure 12). Then, we introduced D96V-CaM into these cells via the patch pipette to determine its impact on I_Na_. Similar to the studies conducted in murine cardiomyocytes, dialysis of D96V-CaM in human iPSC-CMs induced late I_Na_ and a depolarizing shift in voltage-dependent inactivation, which was sensitive to 300 nM 4,9ahTTX (Figure 2, G and H, and Supplemental Figure 13, A and B). These findings support the notion that D96V-CaM may contribute to pathogenic late I_Na_ not only in mice but also in humans.

### D96V-CaM dysregulates Na_V_1.6 but not Na_V_1.5.

**Figure 3:**
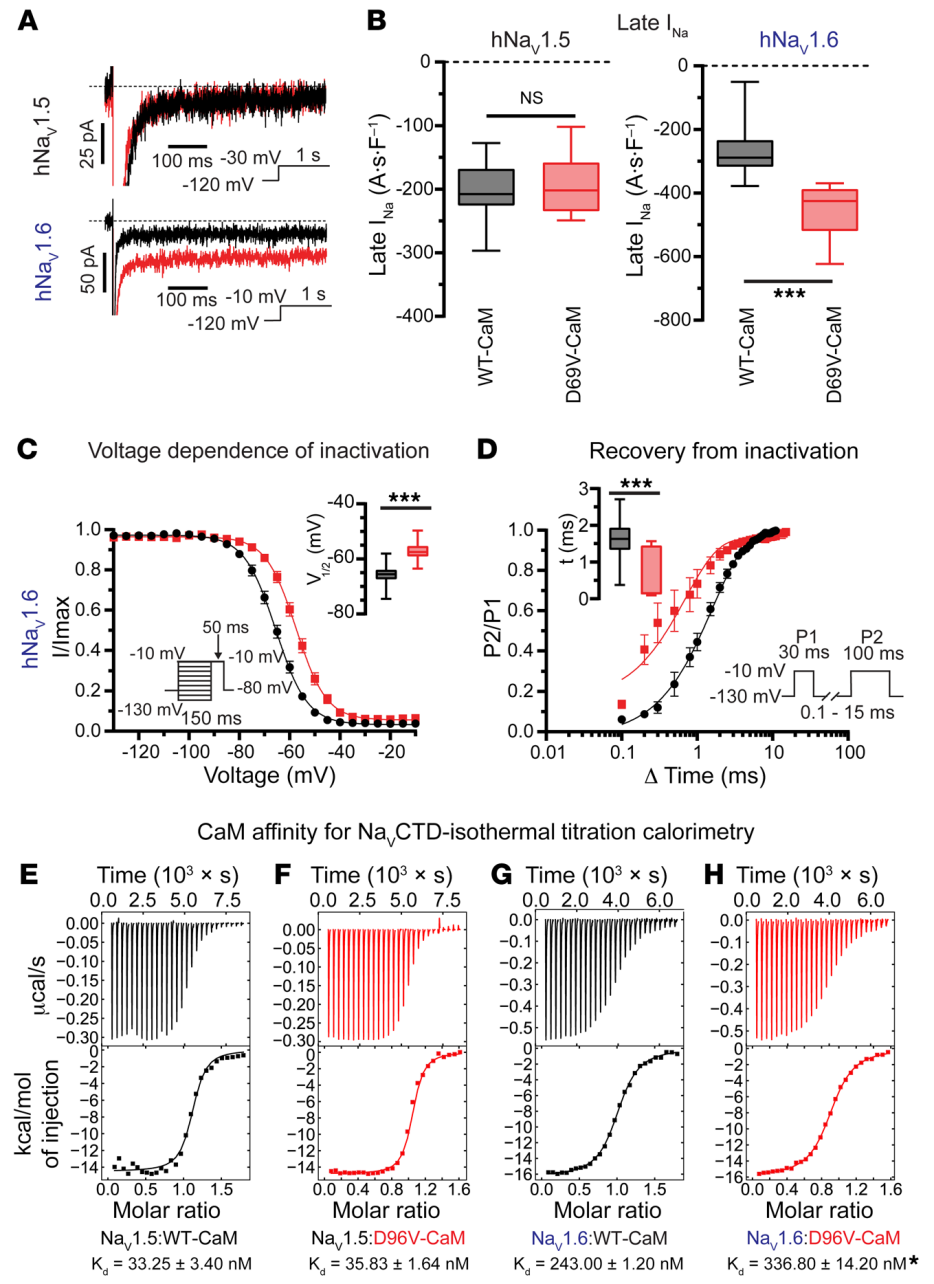
D96V-CaM dysregulates Na_V_1.6 through weakened interaction with Na_V_1.6CTD. (**A**) Representative late I_Na_ traces recorded in CHO cells stably expressing hNa_V_1.5 (top) and hNa_V_1.6 (bottom) that were dialyzed with 6.5 μM WT CaM (black trace) or 6.5 μM D96V-CaM (red trace) and recorded during the voltage-step protocol illustrated under each trace. In these experiments, the recombinant CaMs were not FLAG tagged. (**B**) Summary: Late I_Na_ integral. For WT CaM and D96V-CaM in hNa_V_1.5, *n* = 15 and *n* = 10, respectively. NS, by 2-tailed Student’s *t* test. For WT CaM and D96V-CaM in hNa_V_1.6, *n* = 22 and *n* = 16, respectively. ***P < 0.001, by Mann-Whitney *U* test. (**C**) Voltage dependence of steady-state inactivation (SSI). For WT CaM and D96V-CaM, *n* = 16 and *n* = 14, respectively. ***P < 0.001, by 2-tailed Student’s *t* test. (**D**) Recovery from inactivation. For WT CaM and D96V-CaM, *n* = 13 and *n* = 12, respectively. ***P < 0.001 Mann-Whitney *U* test. (**E**–**H**) Representative ITC measurements at 0 Ca^2+^ of (**E** and **F**) the hNa_V_1.5CTD (residues 1897–1924) and (**G** and **H**) the hNa_V_1.6CTD (residues 1891–1918) binding to WT CaM or D96V-CaM. Raw (top) and cumulative (bottom) plots of the heat evolved following the injections. All curves were fitted to a model of 1 binding site per monomer. *n* = 4 replicates for hNa_V_1.5CTD:WT CaM, hNa_V_1.5CTD:D96V-CaM, and hNa_V_1.6CTD:D96V-CaM; *n* = 2 replicates for hNa_V_1.6:WT CaM. For the *K_D_* of WT CaM:hNa_V_1.6CTD versus D96V-CaM:hNa_V_1.6CTD at 0 Ca^2+^ and 10 μM Ca^2+^. *q < 0.05, by 1-way ANOVA with the original FDR method of Benjamini and Hochberg for post hoc comparisons.

To obtain orthogonal validation of our pharmacological studies in cardiomyocytes, we sought further confirmation of the Na_V_ isoform dysregulated by D96V-CaM using CHO cells stably expressing either human Na_V_1.5 (hNa_V_1.5) or hNa_V_1.6. In line with previous reports (13), dialysis of D96V-CaM into hNa_V_1.5-containing CHO cells did not alter late I_Na_ (Figure 3, A and B) or its activation/inactivation properties relative to WT CaM (Supplemental Figure 14). In contrast, D96V-CaM significantly increased hNa_V_1.6 late I_Na_ relative to WT CaM (Figure 3, A and B), without affecting the density or activation properties of peak I_Na_ (Supplemental Figure 15, A–C). These findings lend further support to the notion that D96V-CaM impairs hNa_V_1.6 inactivation as manifested by a depolarizing shift in voltage-dependent inactivation (Figure 3C), accelerated recovery from inactivation (Figure 3D), and prolonged fast and slow inactivation of I_Na_ (Supplemental Figure 15, D and E).

Recent investigation of Na_V_ interaction with CaM has identified the affinity of CaM for the Na_V_CTD-containing IQ motif as a predictor of late I_Na_ magnitude (11). To gain insight into the affinity of D96V-CaM for Na_V_CTDs, we performed isothermal titration calorimetry (ITC) with 0 Ca^2+^ (Supplemental Table 1). We measured a *K_D_* of 33.25 ± 3.404 nM for the Na_V_1.5-CTD and WT CaM (Figure 3E and Supplemental Table 1), consistent with previous measurements (11, 32). The *K_D_* for D96V-CaM and Na_V_1.5-CTD was similar (*K_D_* = 35.83 ± 1.639 nM; Figure 3F and Supplemental Table 1). In contrast, the *K_D_* for the D96V-CaM and Na_V_1.6-CTD was significantly higher than the *K_D_* for WT CaM and Na_V_1.6-CTD (336.80 ± 14.20 nM vs. 243.00 ± 1.20 nM, respectively; Figure 3, G and H, and Supplemental Table 1). Furthermore, consistent with previous reports (10), ITC measurements performed in the presence of 10 μM free Ca^2+^ (Supplemental Table 1 and Supplemental Figure 16, A and B) demonstrated a reduced *K_D_* for WT CaM and the Na_V_1.6-CTD (132.3 ± 11.020 nM) relative 0 Ca^2+^. Similarly, at 10 μM free Ca^2+^, we observed a reduced *K_D_* for D96V-CaM and the Na_V_1.6-CTD (239.7 ± 27.200 nM), which was higher than the *K_D_* for the WT CaM and Na_V_1.6-CTD under the same conditions. These data support the notion that a reduced affinity of D96V-CaM for Na_V_1.6-CTD may in part contribute to impaired inactivation of Na_V_1.6 by this LQT-associated mutant CaM.

### D96V-CaM promotes structural remodeling of Na_V_1.6 near RyR2.

To examine the arrhythmogenic potential of D96V-CaM, we generated a transgenic mouse with cardiac-specific expression of D96V-CaM (cD96V; TgD96V-CaM β-MHC-Cre). The contractile function of cD96V hearts was normal, independent of age, with no evidence of extensive fibrosis in the older population of mice (Supplemental Figures 17 and 18). Total CaM protein expression in cD96V hearts remained unchanged relative to WT (Supplemental Figure 19; see full-length Western blot of calmodulin in the supplemental material). Next, we confirmed expression of D96V-CaM in cD96V, but not WT, hearts, and its close spatial association with Na_V_1.6 and RyR2 along T-tubules (Supplemental Figures 4 and 6). Together, these data support a close association of RyR2, Na_V_1.6, and D96V-CaM in cD96V myocardium.

**Figure 4:**
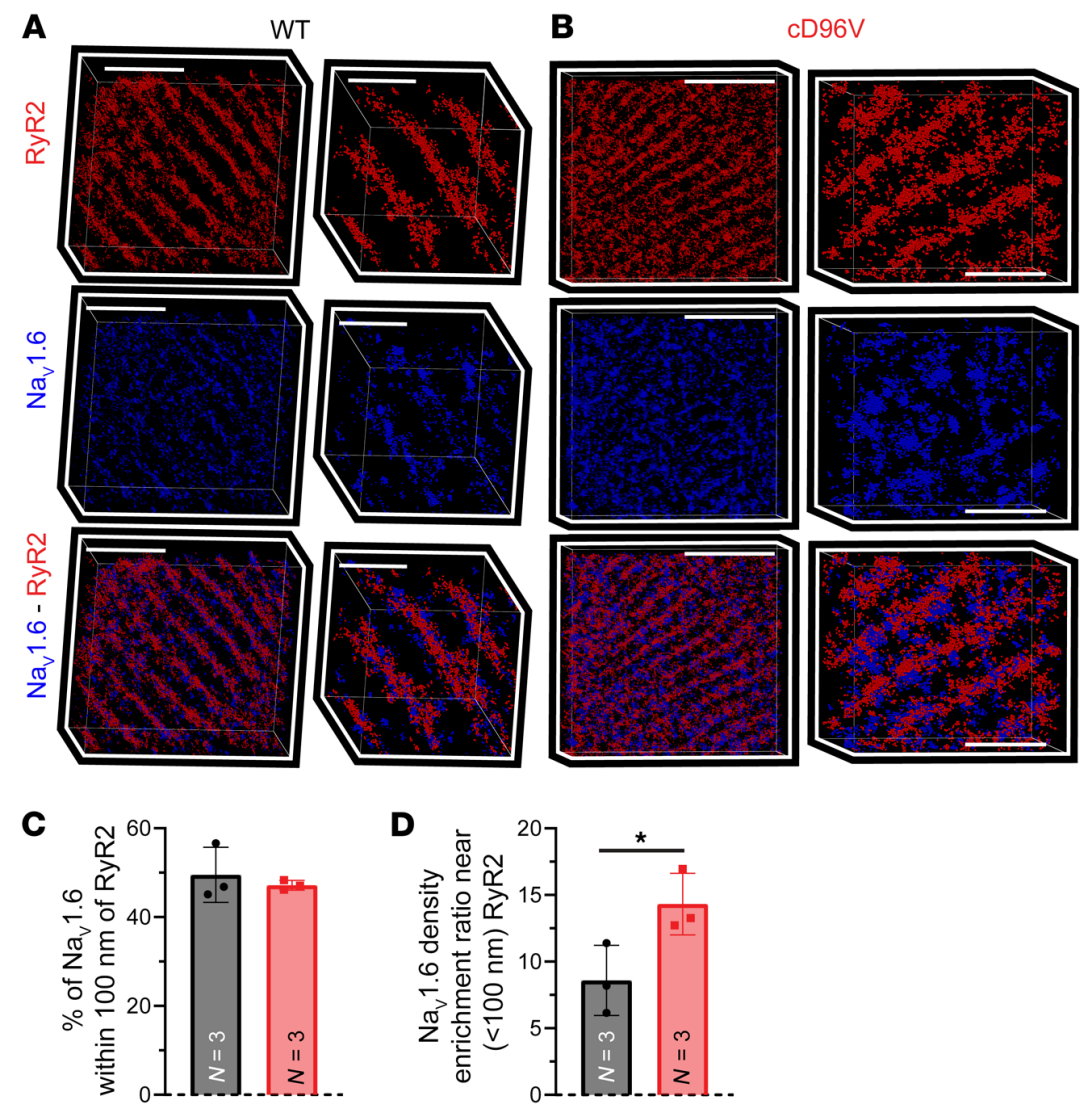
Close proximity (<100 nm) between Na_V_1.6 and RyR2 in cD96V hearts. Representative STORM images (point cloud representation) from (**A**) WT and (**B**) FLAG-tagged cD96V hearts immunolabeled for RyR2 (top, red), Na_V_1.6 (middle, blue), and overlay (bottom) wide views (left) and zoomed-in views (right). Scale bars: 5 μm (left in **A**) and 2 μm (right in **A**); 6 μm (left in **B**) and 2 μm (right in **B**). (**C**) Percentage of Na_V_1.6 located within 100 nm of RyR2 in WT (black) and cD96V (red) myocardium. (**D**) Density of Na_V_1.6 clusters located within 100 nm of RyR2 relative to the ones located farther than 100 nm from RyR2. *n* = 3 replicates for 3 WT mice (*n* = 1 male, *n* = 2 females, 22 weeks old) and 3 cD96V mice (*n* = 1 male, *n* = 2 females, 21–31 weeks old). Data indicate the mean ± SEM. *P < 0.05, by 2-tailed Student’s *t* test.

Next, we used super-resolution stochastic optical reconstruction microscopy (STORM) (20–25 nm resolution) to probe the relationship between Na_V_1.6 and Ca^2+^ cycling machinery at subdiffraction resolution. Qualitatively, the STORM images confirmed a close (<100 nm) association of Na_V_1.6 with RyR2 clusters in both WT and cD96V hearts (Figure 4, A and B). Machine-learning–based cluster analysis (STORM-RLA) (33) revealed that nearly half of the Na_V_1.6 clusters were located within 100 nm of RyR2 in both WT (49.5% ± 3.5%) and cD96V (47.2% ± 0.6%, *P* = NS vs. WT; Figure 4C and Supplemental Figure 20) myocardium. However, the density of these RyR2-adjacent Na_V_1.6 clusters (<100 nm from RyR2) was increased by 67% in cD96V relative to WT myocardium (Figure 4D and Supplemental Figure 20). Together, these results suggest that Na_V_1.6 clusters were closely associated with RyR2-containing junctions within T-tubules, where CaM and LTCCs also reside. This provides a structural substrate for the dysregulation of Na^+^/Ca^2+^ handling within these nanodomains in cD96V hearts, which may promote aberrant Ca^2+^ release that gives rise to arrhythmias in vivo.

### D96V-CaM–mediated Na_V_1.6 dysfunction in T-tubules is associated with abnormal Ca^2+^ release in cD96V cardiomyocytes.

**Figure 5:**
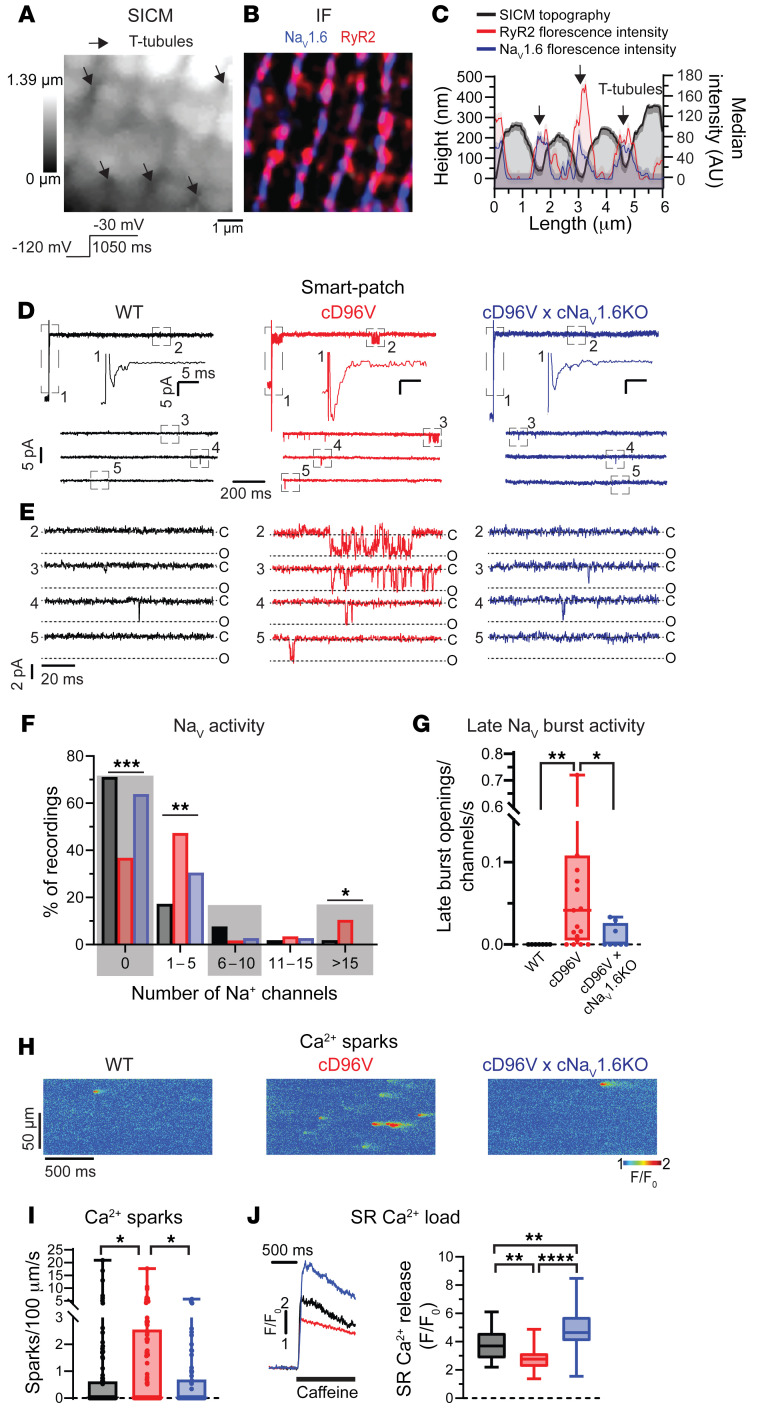
Cardiac-specific expression of D96V-CaM promotes T-tubular late Na_V_1.6 activity and aberrant Ca^2+^ release. (**A**) 3D SICM image of a cardiomyocyte lateral membrane from a WT cardiomyocyte. Arrows indicate T-tubule openings. (**B**) Confocal image of a section of myocardial tissue from a WT mouse (independent sample from **A**) immunolabeled for Na_V_1.6 (blue) and RyR2 (red). Scale bar: 1 μm (**A** and **B**). (**C**) Fluorescence intensity profiles of Na_V_1.6 (blue) and RyR2 (red) from **B** overlaid with a topology profile from **A** correlate Na_V_1.6 and RyR2 signal intensity with T-tubules (arrows). (**D**) SICM-guided “smart” patch I_Na_ recordings from T-tubule openings of WT (left, black), FLAG-tagged cD96V (middle, red), and FLAG-tagged cD96V cNa_V_1.6-KO (right, blue) cardiomyocytes. Uppermost traces show full current recordings obtained during the voltage-step protocol; lower traces show late I_Na_ recordings only (50 ms after the test potential onset). Insets: Dashed rectangles from region 1 (top trace), enlarged. (**E**) Late I_Na_ recordings enlarged from dashed rectangles 2–5 from **D**. (**F**) Histograms of Na_V_ openings recorded from T-tubules (relative to the total number of attempts). For WT *n* = 52 cells from 16 mice (*n* = 10 males, *n* = 6 females, 7–13 weeks old); cD96V, *n* = 57 cells from 19 mice (*n* = 10 males, *n* = 9 females, 11–26 weeks old); and cD96V cNa_V_1.6-KO, *n* = 36 cells from 17 mice (*n* = 7 males, *n* = 10 females, 9–25 weeks old). ***P < 0.001, **P < 0.01, and *P < 0.05, by χ^2^ test. (**G**) Frequency of burst Na_V_ openings (normalized to the number of Na_V_s in membrane patches and the cumulative durations of current registrations). For WT, *n* = 7 cells form 4 mice (*n* = 3 males, *n* = 1 female, 7–10 weeks old); cD96V, *n* = 19 cells from 11 mice (*n* = 4 males, *n* = 7 females, 11–26 weeks old); and cD96V cNa_V_1.6-KO, *n* = 8 cells from 6 mice (*n* = 4 males, *n* = 2 females, 9–25 weeks old). **q < 0.01 and *q < 0.05, by Kruskal-Wallis test with the original FDR method of Benjamini and Hochberg for multiple comparisons. (**H**) Confocal Ca^2+^ sparks recorded in linescan mode from WT (left), cD96V (middle), and cD96V cNa_V_1.6-KO (right) cardiomyocytes paced at 0.3 Hz. (**I**) Ca^2+^ spark frequencies. For WT, *n* = 96 cells from 13 mice (*n* = 7 males, *n* = 6 females, 8–23 weeks old); cD96V, *n* = 106 cells from 10 mice (*n* = 4 males, *n* = 6 females, 10–26 weeks old); and cD96V cNa_V_1.6-KO, *n* = 74 cells from 8 mice (*n* = 5 males, *n* = 3 females, 6–26 weeks old). *q < 0.05, by Kruskal-Wallis test with the original FDR method of Benjamini and Hochberg for multiple comparisons. (**J**) SR Ca^2+^ load measured as Ca^2+^ transient amplitude elicited with 20 mM caffeine (caffeine-induced Ca^2+^ transient). For WT, *n* = 34 cells from 8 mice (*n* = 6 males, *n* = 2 females, 9–23 weeks old); cD96V, *n* = 30 cells from 12 mice (*n* = 4 males, *n* = 8 females, 8–25 weeks old); and cD96V cNa_V_1.6-KO, *n* = 27 cells from 6 mice (*n* = 3 males, *n* = 3 females, 14–26 weeks old). **q < 0.01 and ****q < 0.0001, by ordinary 1-way ANOVA with the original FDR method of Benjamini and Hochberg for multiple comparisons.

Next, we examined the pathophysiological consequences of D96V-CaM–mediated Na_V_1.6 dysfunction and remodeling near RyR2 in cD96V cardiomyocytes. Since a whole-cell patch clamp provides a lumped, cell-wide assessment of Na^+^ flux, we performed scanning ion conductance microscopy–guided (SICM-guided) “smart” patch-clamp experiments to gain insight into local dysfunction of Na_V_s within Ca^2+^ cycling nanodomains. A smart patch clamp enables the recording of single-channel activity in the cell-attached configuration from T-tubules localized based on membrane topography (Figure 5A) (34). Correlative analysis of SICM and confocal immunofluorescence data supported the coincidence of T-tubules with Na_V_1.6 and RyR2 (Figure 5, A–C). Smart patch recordings from T-tubule regions revealed Na_V_ activity in a significantly higher proportion of membrane patches in cD96V cardiomyocytes relative to WT cardiomyocytes (Figure 5, D–F). Importantly, T-tubules in cD96V cardiomyocytes evidenced a significantly higher probability of large Na_V_ clusters (>15 channels) and a larger proportion of late Na_V_ burst openings relative to WT cardiomyocytes (Figure 5, F and G, and Supplemental Figure 2), consistent with enhanced whole-cell late I_Na_ (Figure 2, A and B). Importantly, the enlargement of Na_V_ clusters and their late activity were abrogated (Figure 5, F and G) upon the crossing of cD96V mice with our previously validated cardiac-specific Na_V_1.6-KO mice (35) (cD96V cNa_V_1.6-KO; Supplemental Figures 4 and 5), confirming the isoform identity of Na_V_s responsible for pathological late Na_V_ activity in cD96V. Together, these findings indicate that D96V-CaM enhances Na^+^ influx into T-tubules by dysregulating Na_V_1.6 inactivation and increasing the number of channels within the Na_V_1.6 clusters.

We next examined whether increased Na_V_1.6 activity within T-tubules of cD96V myocytes promoted Ca^2+^ sparks. Confocal Ca^2+^ imaging demonstrated a significant increase in the frequency and amplitude of Ca^2+^ sparks in cD96V cells relative to WT and cD96V cNa_V_1.6-KO cells (Figure 5, H and I, and Supplemental Figure 21) without affecting Ca^2+^ spark duration or width (Supplemental Figure 21). The enhanced Ca^2+^ release was coupled to a reduction in sarcoplasmic reticulum (SR) Ca^2+^ load in cD96V cells relative to WT cells (Figure 5J). In line with previous findings, which suggest that such aberrant Ca^2+^ release may be a consequence of Na^+^-Ca^2+^ exchange (21–23, 36), Na_V_1.6 was closely associated with Na^+^-Ca^2+^ exchanger (NCX) in both WT and cD96V hearts (Supplemental Figure 22). Of note, the SR Ca^2+^ load in cD96V cNa_V_1.6-KO cardiomyocytes was higher than in cD96V or even WT cardiomyocytes. This may reflect the combined effect of fewer aberrant Ca^2+^ release events coupled with an increased Ca^2+^ influx secondary to dysregulated LTCCs (7, 13, 28). Both cD96V and cD96V cNa_V_1.6-KO showed compromised I_Ca_ inactivation relative to WT hearts (Supplemental Figure 23), confirming that Ca^2+^ loading through dysregulated LTCCs contributed to an enhanced SR Ca^2+^ load (Figure 5J) and APD prolongation (Supplemental Figure 11) in these models. Together, these findings suggest that dysregulation of both Na_V_1.6 and LTCCs contributes to APD prolongation and abnormal Ca^2+^ handling in D96V-associated calmodulinopathy.

### D96V-CaM–mediated dysregulation of Na_V_1.6 promotes Ca^2+^-dependent arrhythmias.

**Figure 6:**
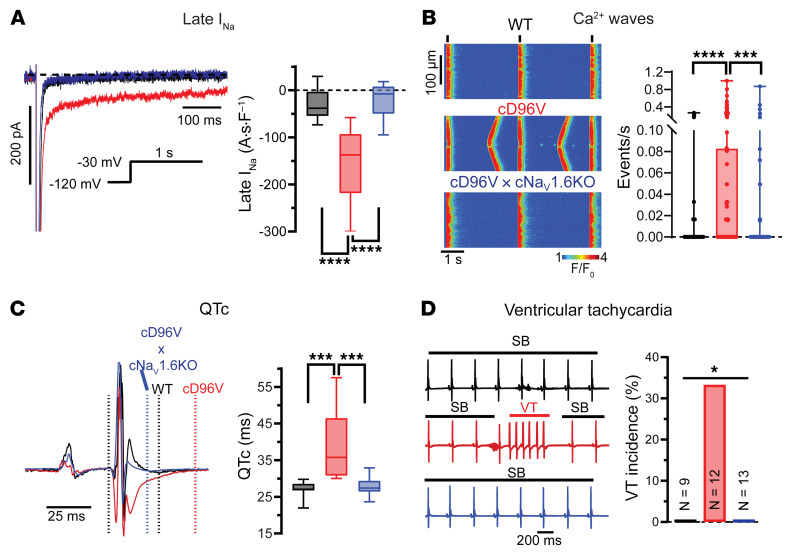
Cardiac-specific expression of D96V-CaM promotes Na_V_1.6-mediated cellular and in vivo arrhythmias. (**A**) Late I_Na_ measurements. For WT, *n* = 13 cells from 6 mice (*n* = 3 males, *n* = 3 females, 6–19 weeks old); FLAG-tagged cD96V, *n* = 9 cells from 4 mice (*n* = 2 males, *n* = 2 females, 20–25 weeks old); and FLAG-tagged cD96V cNa_V_1.6-KO, *n* = 15 cells from 5 mice (*n* = 2 males, *n* = 3 females, 7–25 weeks old). ****q < 0.0001, by ordinary 1-way ANOVA with the original FDR method of Benjamini and Hochberg for multiple comparisons. (**B**) Ca^2+^ waves from WT (top), cD96V (middle), and cD96V cNa_V_1.6-KO (bottom) cardiomyocytes paced at 0.3 kHz. Black lines above the image denote stimuli. For WT, *n* = 96 cells from 13 mice (*n* = 7 males, *n* = 6 females, 9–27 weeks old); cD96V, *n* = 106 cells from 10 mice (*n* = 6 males, *n* = 4 females, 9–31 weeks old); and cD96V cNa_V_1.6-KO, *n* = 92 cells from 8 mice (*n* = 6 males, *n* = 2 females, 6–31 weeks old). ***q < 0.001 and ****q < 0.0001, by Kruskal-Wallis test with the original FDR method of Benjamini and Hochberg for multiple comparisons. (**C**) Surface ECGs and QTc intervals from WT, cD96V, and cD96V cNa_V_1.6-KO mice. For WT, *n* = 9 mice (*n* = 4 males, *n* = 5 females, 12–25 weeks old); cD96V, *n* = 12 mice (*n* = 7 males, *n* = 5 females, 6–18 weeks old); and cD96V cNa_V_1.6-KO, *n* = 13 mice (*n* = 9 males, *n* = 4 females, 6–26 weeks old). ***q < 0.001, by Kruskal-Wallis test with the original FDR method of Benjamini and Hochberg for multiple comparisons. (**D**) ECGs recorded from mice after carbachol challenge (0.5 mg/kg i.p. injection), which resulted in sinus bradycardia (SB) (heart rate <200 beats per minute). VT was only observed in 4 of 12 cD96V mice. *P < 0.05, by χ^2^ test.

To examine the cellular arrhythmia potential of D96V-CaM–mediated Na_V_1.6 dysregulation within T-tubules, we examined macroscopic cell-wide Na^+^ and Ca^2+^ handling properties. Whole-cell patch-clamp recordings revealed an increase in late I_Na_ in cD96V relative to WT cardiomyocytes (Figure 6A), which was accompanied by a depolarizing shift in voltage-dependent inactivation (Supplemental Figure 24, A and B) without significant changes in peak I_Na_ properties (Supplemental Figure 24, C–E). Notably, cardiac-specific Na_V_1.6 KO in cD96V (cD96V cNa_V_1.6-KO) mice ameliorated late I_Na_ (Figure 6A) and restored voltage-dependent I_Na_ inactivation (Supplemental Figure 24A), while reducing peak I_Na_ by 31.53% ± 5.43% relative to cD96V (Supplemental Figure 23C). We next compared this reduction in I_Na_ with that achieved by 300 nM 4,9ahTTX (Supplemental Figure 25). In cD96V cardiomyocytes, we found that 4,9ahTTX significantly and reversibly suppressed late and peak I_Na_, with the latter being reduced by 34.76% ± 5.84%. However, in cD96V cNa_V_1.6-KO cardiomyocytes, 4,9ahTTX did not produce an effect (Supplemental Figure 25), suggesting that at this concentration, 4,9ahTTX exerted a negligible effect on other TTX-sensitive Na_V_ isoforms in this murine model. Since 4,9ahTTX has previously been shown to inhibit a fraction of Na_V_1.1 (37), we then compared the extent of I_Na_ reduction obtained with 4,9ahTTX with that elicited by a recently developed Na_V_1.6 blocker, NBI-921352 (38). In cD96V, we observed that NBI-921352 (1 μM) produced a reduction of late and peak I_Na_ (32.52% ± 6.25%) similar to that achieved with 4,9ahTTX (300 nM) or observed in cD96V cNa_V_1.6-KO cardiomyocytes (Supplemental Figure 25).

Confocal Ca^2+^ imaging demonstrated that cD96V cardiomyocytes experienced more frequent Ca^2+^ waves relative to WT cardiomyocytes, which were reduced in cD96V cNa_V_1.6-KO hearts (Figure 6B). Alterations in CaM function could also indirectly modulate Na^+^/Ca^2+^ handling through Ca^2+^/CaM-dependent protein kinase II (CaMKII). However, CaMKII inhibition with myristoylated autocamtide-2–related inhibitor peptide (AIP) (10 μM) did not reduce the incidence of afterdepolarizations in cD96V cardiomyocytes (Supplemental Figure 26), corroborating previous finding that D96V-CaM did not significantly alter CaMKII activity relative to WT CaM (4).

Last, we examined the effects of D96V-CaM–mediated Na_V_1.6 dysregulation on VT inducibility. To this end, we performed surface ECGs in mice undergoing a bradycardia challenge with carbachol (0.5 mg/kg, i.p. injection) to mimic the bradycardic conditions that promote LQT-associated VT in patients (39). At baseline, consistent with the LQT phenotype, cD96V mice showed a prolonged QT/QTc interval compared with WT mice, which was mitigated by cD96V cNa_V_1.6-KO (Figure 6C and Supplemental Figure 9). Notably, the magnitude of QT/QTc prolongation in cD96V mice was similar to that observed with another LQT-associated CaM mutant, N98S-CaM (8). Importantly, the bradycardic challenge induced VT in 4 of 12 cD96V mice, regardless of age (1 incidence in 6- to 8-week-old mice; 2 in 8- to 17-week-old mice; and 1 in 18- to 19-week-old mice), but not in any of the WT or cD96V cNa_V_1.6-KO mice tested (Figure 6D). In all, the D96V-CaM dysregulation of Na_V_1.6 corroborated the emerging role of LQT-associated CaM in the modulation of arrhythmogenic late I_Na_ in the heart.

## Discussion

**Figure 7:**
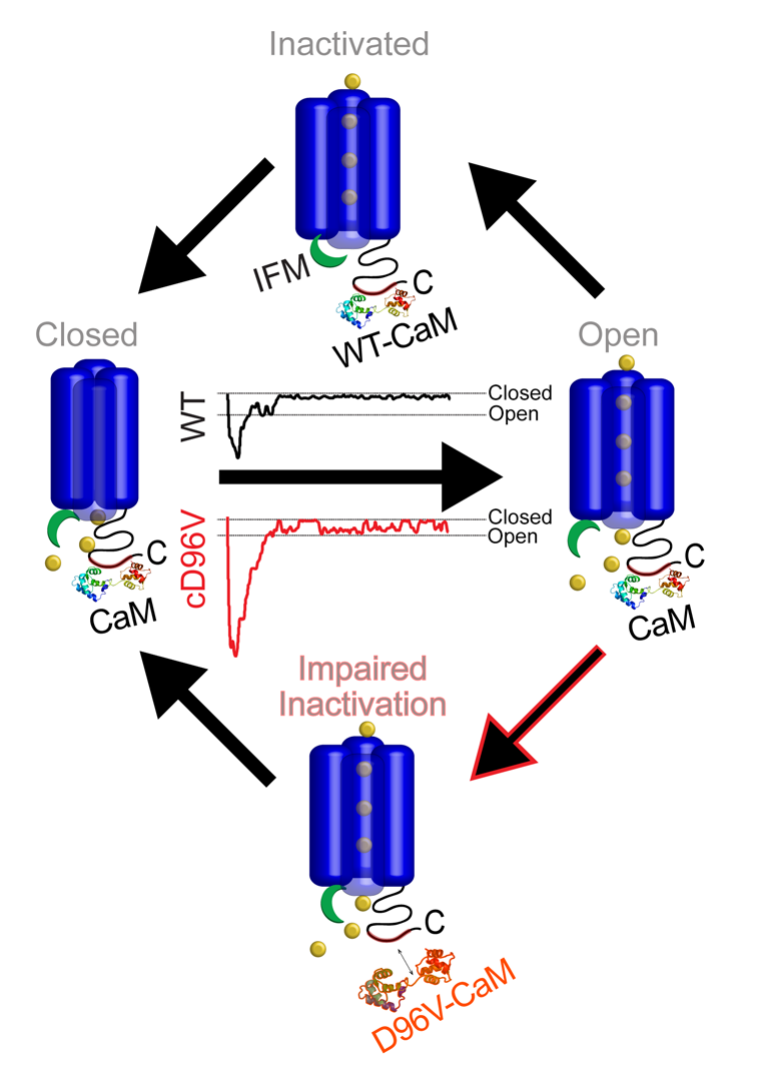
Effect of D96V-CaM on Na_V_1.6 inactivation and late Na_V_ activity. Reduced interaction between D96V-CaM and Na_V_1.6-CTD destabilizes Na_V_1.6 inactivation, resulting in late Na_V_1.6 burst activity.

Arrhythmogenic CaM mutants have been linked to impaired function of Ca^2+^-handling proteins, resulting in LQT syndrome, catecholaminergic polymorphic VT, or idiopathic ventricular fibrillation (1). However, to date, no clear link has been recognized between CaM mutations, Na_V_ dysfunction, and arrhythmias (8, 13, 14, 40). Here, we identify, for the first time to our knowledge, a LQT-associated CaM mutation (D96V-CaM) that exhibits reduced interaction with Na_V_1.6, thereby impairing channel inactivation (Figure 7). Unexpectedly, we found that D96V-CaM–mediated Na_V_ dysfunction was coupled to Na_V_1.6 nanodomain cluster remodeling within cardiac T-tubules. This functional remodeling of Na_V_s, along with a well-described slowed LTCC inactivation (7, 28), promotes aberrant Ca^2+^ release at the cellular level and cardiac arrhythmias in vivo.

### D96V-CaM impairs Na_V_1.6 inactivation.

LQTS-associated CaM mutants impair LTCC Ca^2+^-dependent inactivation, which has been linked to APD prolongation (7), a result that we corroborate here (Supplemental Figure 23). Furthermore, it has been hypothesized that a similar dysregulation of Na_V_s may promote proarrhythmic late I_Na_. However, previous investigations, which focused on Na_V_1.5, the most abundant Na_V_ isoform in the heart, proved inconclusive (13, 14). Specifically, D130G- and E141G-CaM mutants have been linked to impaired Na_V_1.5 inactivation in heterologous systems under specific conditions (13, 14), although their roles under native conditions in the heart remain unclear. Additionally, investigations in human iPSC-CMs expressing *CALM1*-F142L and murine hearts expressing *Calm1*-N98S failed to demonstrate enhancement of late I_Na_ (8, 40). We present, to our knowledge, the first results from single-molecule through in vivo scales demonstrating a role for mutant CaM–mediated (D96V-CaM–mediated) impaired inactivation of a Na_V_ in arrhythmias. Notably, D96V-CaM promoted proarrhythmic late I_Na_ via T-tubule–localized Na_V_1.6 channels rather than the more abundant Na_V_1.5 channels.

The Na_V_CTD, containing an IQ motif that enables CaM interaction, has emerged as a hotspot for mutations associated with arrhythmias (41). Outside of the arrhythmogenic mutations in Na_V_1.5CTD, recent findings directly link mutations in Na_V_1.6CTD with early infantile epilepsy [NM_001330260.2(SCN8A):c.5710C>T; p.Arg1904Cys] (42), while early reports of Na_V_1.6 gain-of-function mutations have described effects on cardiac electrophysiology (22, 43). Intriguingly, mutations in Na_V_1.6CTD at the CaM binding region (R1902, Y1904, and R1905) destabilize CaM-Na_V_1.6CTD interaction and thereby impair Na_V_1.6 inactivation (10). Consistent with this notion, mutations in Na_V_1.5 and Na_V_1.2 CTDs, which reduce CaM affinity, also enhance late I_Na_ (11, 44). Of note, the WT Na_V_1.6 CTD showed the lowest CaM affinity relative to other Na_V_s and exhibited the greatest magnitude of late I_Na_, even when compared with the Na_V_1.5 and Na_V_1.2CTD mutants (11). Akin to work on mutant CaMs in LTCC regulation (45), the reduced affinity of a dominant-negative D96V-CaM mutant for Na_V_1.6CTD may not fully explain the impact of this mutation on Na_V_1.6 inactivation. Future work will need to examine the cooperativity of additional CaM binding sites and other Na_V_-interacting proteins, such as FGF homologous factors (46), with the Na_V_CTD on Na_V_ inactivation. However, our ITC findings, which point toward the potential proarrhythmic properties of Na_V_1.6, especially when dysregulated, provide a useful context for our results implicating the channel in D96V-CaM–mediated arrhythmias.

### D96V-CaM–mediated increase in late Na_V_1.6 burst activity.

Assessment of whole-cell I_Na_, as discussed above, lacks information on local dysfunction within Na^+^/Ca^2+^-handling nanodomains. To overcome this, we used an SICM-guided “smart” patch clamp to record channel activity from T-tubule openings. We observed an increase in the frequency of late Na_V_1.6 burst openings in cD96V cardiomyocytes relative to WT cardiomyocytes (Figure 5G and Supplemental Figure 2). This nanodomain behavior is consistent with early observations that the frequency of Na_V_ burst openings predicted the magnitude of late I_Na_ in failing myocardium (47). Additionally, the increased burst activity observed in Na_V_1.5 ΔKPQ was sufficient to result in late I_Na_, APD prolongation, and EADs (48). From the biophysical standpoint, the burst mode of a Na_V_ corresponds to transient failure of the channel to inactivate (48). Structurally, such deficient inactivation results from impaired interaction of the domain III–IV linker, another site of CaM-Na_V_ interaction, with the channel pore (49). Changes in the structure of Na_V_CTD are also known to augment single-channel burst activity. Specifically, deletion of the CaM-binding domain of the Na_V_CTD significantly enhanced burst activity and late I_Na_ (50). On the other hand, CaM has been shown to modulate Na_V_1.5- and Na_V_1.6-mediated late I_Na_ by stabilizing the interaction between the proximal part of the Na_V_CTD and the domain III–IV linker (49). Our data, along with emerging research on Na_V_CTD interaction with the domain III–IV linker (49, 51–53), strongly suggest that modifying CaM interaction with Na_V_CTD may impair Na_V_ inactivation by altering crosstalk with the inactivation gate.

### Role of D96V-CaM–mediated Na_V_1.6 dysregulation in cardiac Ca^2+^ mishandling.

Na_V_1.6 carries only a fraction of whole-cell peak I_Na_, as assessed pharmacologically and genetically (Figure 2E and Supplemental Figure 24C). Despite that, increased Na_V_1.6 late burst activity within T-tubules coupled with the proximity of Na_V_1.6 to Ca^2+^-handling proteins (NCX and RyR2) point to the potential influence that Na_V_1.6 dysregulation may exert on Ca^2+^ handling. Consistent with this notion, a D96V-CaM–mediated increase in Na_V_1.6 cluster size and late burst activity within T-tubular nanodomains, along with dysfunctional LTCCs, precipitated an increase in Ca^2+^ sparks (Figure 5, H and I, and Supplemental Figure 21). This wasteful Ca^2+^ release reduced the SR Ca^2+^ load (Figure 5J). Importantly, cardiac-specific KO of Na_V_1.6 ameliorated the Na^+^ mishandling and aberrant Ca^2+^ release observed in cD96V cardiomyocytes, preventing the depletion of SR Ca^2+^ stores. In fact, cD96V cNa_V_1.6-KO unmasked the increased SR Ca^2+^ load, most likely a consequence of increased Ca^2+^ influx through LTCCs (Supplemental Figure 23). These results further confirm the role of hyperfunctional Na_V_1.6 in aberrant Ca^2+^ release. Taken together, D96V-CaM–mediated Na_V_1.6 dysfunction within T-tubules can conspire with dysfunctional LTCCs to promote aberrant Ca^2+^ release.

### Arrhythmogenic mechanism of D96V-CaM–mediated Na_V_1.6 dysregulation.

The abnormally enhanced Na^+^/Ca^2+^ handling observed in cD96V contributed to APD prolongation on the cellular level and QTc prolongation in vivo. This translated into in vivo arrhythmias during bradycardia, a condition associated with LQT-dependent arrhythmias in patients (39). In this respect, our cD96V LQT mouse model parallels a recently described model of N98S-CaM–mediated catecholaminergic polymorphic VT (8). In both models, bradycardic conditions elicited EADs. However, unlike isoproterenol-induced DADs in the N98S-CaM model, DADs with D96V-CaM occurred spontaneously without any additional pharmacological augmentation of Ca^2+^ cycling. This finding supports the notion that D96V-CaM promotes arrhythmias via intrinsic dysregulation of Na^+^/Ca^2+^ handling rather than by simply modulating the response to extrinsic factors such as β-adrenergic–mediated stimulation. In summary, we provide a mechanistic basis for D96V-CaM–mediated dysregulation of Na_V_1.6 inactivation, localization, and activity and link these with arrhythmogenic effects.

### Limitations.

Both acute introduction (dialysis) and transgenic expression of CaM may raise myocyte CaM levels to nonphysiological levels, which may accentuate the effect of D96V-CaM on Na_V_1.6. However, both the cD96V, with CaM expression comparable to that of WT (Supplemental Figure 19; see full-length Western blot of calmodulin in the supplemental material), and dialysis of WT myocytes with a 5-fold lower D96V-CaM concentration, resulted in similar degrees of I_Na_ dysfunction (Figure 2, A–F). These results suggest that, despite the nonphysiologic properties of some models used in this study, the findings may actually reflect the dominant-negative nature of D96V-CaM–mediated I_Na_ dysfunction in calmodulinopathy. Future studies using knock-in approaches will need to confirm our findings in a more physiological milieu. Additionally, because of the proximity of Na_V_1.6 to RyR2 (Figure 4 and Supplemental Figures 6 and 7), Na_V_1.6 may experience a high degree of fluctuation in Ca^2+^ concentrations. In the light of previous work suggesting dependence of Na_V_ inactivation stability on CaM and Ca^2+^ (52, 54, 55), future studies will need to establish whether D96V-CaM interaction with Na_V_1.6 is affected by Ca^2+^ and the functional implications thereof. Furthermore, outside of CaM-Na_V_ interaction affecting Na_V_ function, it has been shown that altered CaM-Na_V_ interaction can affect Na_V_ cell-surface expression (56). In our study, we observed enlarged Na_V_1.6 clusters in T-tubules (Figure 4). In the context of previous findings implicating CaM in the coordination of Kv7 trafficking (57) and Ca_V_1.2 internalization (58), future studies will need to establish the mechanism by which D96V-CaM affects Na_V_1.6 surface expression and clustering.

## Methods

Additional details are provided in the Supplemental Methods.

### Mouse models.

Mice of both sexes, aged 6–31 weeks, were used in the study. WT mice on a C57BL/6 background were purchased from The Jackson Laboratory (catalog 000664). Cardiac-specific Na_V_1.6-KO mice (cNa_V_1.6-KO) were obtained by crossing C57BL/6 mice with loxP sites flanking exon 1 of the *Scn8a* gene (custom generated by the Transgenic and Gene Targeting Core and Mutation Generation and Detection Core at the University of Utah, Salt Lake City, Utah, USA), as described previously (35), with transgenic mice harboring Cre under the cardiac-specific β-myosin heavy chain (Myh7) promoter [Tg(Myh7-cre)1Jmk], a gift from Federica Accornero (The Ohio State University, Columbus, Ohio, USA).

A plasmid containing D96V-CaM fused with a C-terminal FLAG tag (pRP[Exp]-CAG>{mCam I*(D96V)}/FLAG:IRES:mCherry) was custom generated by Cyagen. The plasmid contained the mutated mouse CaM gene (CaM I) CDS (GenBank: M19381.1, CDS 187.633, mutation: 473A > T) with a FLAG-tagged DNA sequence in the same ORF. The D96V-CaM/FLAG ORF was separated from the upstream CAG promoter by the floxed 3 × CV40 pA sequence in order to allow for Cre-dependent expression of D96V-CaM/FLAG. Mice with global incorporation of the D96V-CaM–floxed construct on a C57BL/6 background were custom generated by Cyagen. Cardiac-specific expression of D96V-CaM (cD96V) was achieved by crossing D96V-CaM–floxed mice with β-myosin heavy chain Cre expressed on a predominantly C57BL/6 background. We crossed cD96V mice with cNa_V_1.6-KO mice to generate cD96V mice with cardiac-specific Na_V_1.6 (cD96V cNa_V_1.6-KO). We confirmed on both mRNA and protein levels that cD96V hearts expressed Na_V_1.6 and Na_V_1.5 at levels similar to WT (Supplemental Figure 1; see full-length Western blot of Na_V_1.6 and Na_V_1.5 in the supplemental material).

### Recombinant CaM and Na_V_CTD peptides.

WT and D96V-CaM were expressed in *E*. *coli* and purified as previously described (59). The Na_V_CTD peptides corresponding to residues 1897–1924 of human Na_V_1.5 (RRKHEEVSAMVIQRAFRRHLLQRSLKHA) and to residues 1891–1918 of human Na_V_1.6 (RRKQEEVSAVVLQRAYRGHLARRGFICK) were synthesized by LifeTein. Peptides of at least 90% purity confirmed by HPLC were used in the study.

### Isothermal titration calorimetry.

Experiments were performed on a Microcal VP-ITC (Malvern Instruments) at 25°C in buffer containing 10 mM Mops, 2 mM EGTA, 1 mM TCEP, and 150 mM KCl, pH 7. Na_V_1.5CTD (55–75 μM) or Na_V_1.6CTD (73–110 μM) peptides were titrated with WT CaM or D96V-CaM (5–15 μM). Titrations were performed with 28 injections, 1 of 5 μL and 27 of 10 μL, with a 4-minute interval between injections. Raw thermograms were processed using NITPIC (60), and isotherms were fitted using SEDPHAT (61) and visualized using GUSSI (62) (all programs from The University of Texas Southwestern Medical Center, Dallas, Texas, USA).

### Murine cardiomyocyte isolation and tissue collection.

Ventricular cardiomyocytes were isolated as previously described (35, 63). Briefly, mice were anesthetized with 5% isoflurane mixed with 100% oxygen (1 L/min), and once a deep level of anesthesia was confirmed, hearts were rapidly excised and submerged in cold Ca^2+^-free Tyrode’s solution consisting of 133.5 mM NaCl, 4 mM KCl, 1 mM MgCl_2_, 10 mM glucose, and 10 mM HEPES, and the pH was adjusted to 7.4 with NaOH. Subsequently, the aorta was cannulated using a blunt 24 gauge needle, transferred to a Langendorff apparatus, and perfused with Ca^2+^-free Tyrode’s solution at 37°C to wash out the remaining blood. Next, the heart was perfused with Ca^2+^-free Tyrode’s solution containing Liberase TH (Roche). The heart was then removed from the perfusion system, and the ventricles were isolated from the atria, minced in Tyrode’s solution containing 2% BSA (MilliporeSigma), dispersed by gentle agitation, and filtered through a nylon mesh. Cardiomyocytes were then resuspended in low-Ca^2+^ Tyrode’s solution consisting of 133.5 mM NaCl, 4 mM KCl, 1 mM MgCl_2_, 0.1 mM CaCl_2_, 10 mM glucose, and 10 mM HEPES, and the pH was adjusted to 7.4 with NaOH. Cells were stored at room temperature and used within 5 hours of isolation. For experiments, cells were plated on laminin-coated coverslips (MilliporeSigma). For subsequent immunolabeling, cells were fixed with 2% paraformaldehyde (PFA) (5 min at room temperature). For studies of mouse myocardial tissues, the excised hearts were frozen in optimal cutting temperature medium (MilliporeSigma) for cryosectioning as previously described (35, 64).

### Cell lines.

Human iPSC-CMs, differentiated from reprogrammed fibroblasts obtained from a healthy male (Axol Biosciences) were cultured on fibronectin-coated glass coverslips and maintained in Cardiomyocyte Maintenance Medium (Axol Biosciences) for 14 days, after thawing before the start of experiments. For immunolabeling, cells were fixed in 2% PFA (5 min at room temperature).

CHO cell lines stably expressing hNa_V_1.5 or hNa_V_1.6 (B’SYS GmbH) were cultured in F-12 medium with glutamine (Thermo Fisher Scientific) supplemented with 10% (v/v) FBS (MilliporeSigma) and 1% penicillin-streptomycin solution at 10,000 U/mL (Thermo Fisher Scientific). G-418 sulphate (500 μg/mL) and 2 μg/mL puromycin (Thermo Fisher Scientific) were added to the medium to select for CHO-hNa_V_1.5, and 500 μg/mL hygromycin (Thermo Fisher Scientific) was used to select CHO-hNa_V_1.6. Cells were cultured at 37°C in 5% CO_2_ in a humidified atmosphere.

### Patch-clamp recordings of APs and confocal Ca^2+^ imaging.

Simultaneous recordings of patch-clamp APs and Ca^2+^ release were performed as previously described (29). Briefly, recordings in cardiomyocytes were performed with an Axopatch 200B amplifier and Digidata 1440A (Molecular Devices) and using an external solution that contained 140 mM NaCl, 5.4 mM KCl, 1.8 mM CaCl_2_, 0.5 mM MgCl_2_, 10 mM HEPES, and 5.6 mM glucose, pH 7.4. Patch pipettes were filled with a solution that contained 90 mM potassium aspartate, 50 mM KCl, 5 mM MgATP, 5 mM NaCl, 1 mM MgCl_2_, 0.1 mM Tris GTP, 10 mM HEPES, and 0.1 mM EGTA, pH 7.2. For simultaneous Ca^2+^ imaging, the pipette solution was supplemented with 0.03 mM Fluo-3 pentapotassium (Molecular Probes). The total CaM level within cardiomyocytes is approximately 6 μM (65), whereas the free CaM level is approximately 100 nM (66). Since CaM is constitutively bound to Na_V_s and it is bound CaM rather than free CaM that regulates Na_V_ function, we attempted to displace the native CaM in cells by introducing 6.5 μM exogenous WT CaM or D96V-CaM, except in some experiments, as noted. APs were evoked by injection of a brief stimulus current of 1.5–4 nA for 0.5–1 ms. Myocytes were paced at 0.3 Hz to obtain a Ca^2+^ wave frequency. Concurrently, linescan imaging of intracellular Ca^2+^ was performed using a Nikon A1R laser-scanning confocal microscope equipped with a 60× 1.4NA oil-immersion objective under 488 nm excitation, and emitted light was collected at 500–600 nm. Any aberrant Ca^2+^ release event (i.e., a wave or wavelet) that increased cell-wide fluorescence intensity by more than 10% of the signal generated by the preceding stimulated Ca^2+^ transient was included in the analysis. The fluorescence emitted is expressed as *F*/*F*_0_, where *F* is the fluorescence at time *t* and *F*_0_ represents the background signal. An EAD was defined as a transient slowing or reversal of the membrane potential during repolarization, whereas a DAD was defined as a positive fluctuation from the resting membrane potential of more than 4 mV. After baseline recordings, we applied 4,9ahTTX (300 nM, Focus Biomolecules). All experiments were performed at room temperature (~26°C).

### Whole-cell patch-clamp recordings of I_Na_.

Current recordings in voltage clamp configuration were made with a MultiClamp 700B amplifier and Digidata 1440A (Molecular Devices). For mouse cardiomyocytes, CHO cells, and human iPSC-CMs, late and peak Na^+^ currents (I_Na_) were recorded using pipette solution containing 10 mM NaCl, 20 mM TEACl, 123 mM CsCl, 1 mM MgCl_2_, 0.1 mM Tris-GTP, 5 mM MgATP, 10 mM HEPES, and 10 mM EGTA to maintain free Ca^2+^ at approximately 0 nM (in experiments with CaM dialysis), or 1 mM EGTA and 0.35 mM CaCl_2_ to maintain free Ca^2+^ at approximately 100 nM (in experiments without CaM dialysis) at pH 7.2 (adjusted with CsOH). Cells were equilibrated for 5 minutes after patching. For CaM dialysis experiments (28), the internal solution was supplemented with WT CaM (6.5 μM) or D96V-CaM (6.5 μM), unless otherwise stated, and cells were equilibrated for 20 minutes. For I_Na_ recordings, the extracellular bathing solution contained 140 mM NaCl, 4 mM CsCl, 1 mM CaCl_2_, 2 mM MgCl_2_, 0.05 mM CdCl_2_, 10 mM HEPES, 10 mM glucose, 0.03 mM niflumic acid, and 0.004 mM strophanthidin, and the pH was maintained at 7.4. For peak I_Na_ recordings in mouse cardiomyocytes, the extracellular bathing solution was altered by reducing NaCl to 10 mM, increasing CsCl to 123 mM, and adding 20 mM TEACl. In experiments with 4,9ahTTX (Focus Biomolecules), currents were recorded 5 minutes after drug application. Patch pipettes had a resistance of 1.2–1.6 MΩ after heat polishing. Compensation for whole-cell capacitance and series resistance (≥60%) was applied along with leak subtraction. Signals were filtered with a 10 kHz Bessel filter, and I_Na_ was then normalized to the membrane capacitance.

Currents were analyzed with the Clampfit module of pClamp, version 10 (Molecular Devices). Late I_Na_ was estimated by integrating I_Na_ between 50 and 450 ms from the test potential onset and normalized to the cell capacitance (29). Experimentally measured steady-state inactivation and activation parameters for I_Na_ were fitted with a sigmoidal Boltzmann function, and the time course of recovery from inactivation was fitted with a single exponential function. The peak I_Na_ decay phase was fitted with the 2-exponential function.

### Scanning ion conductance microscopy–guided smart patch clamp.

The SICM-guided smart patch clamp is a noncontact scanning probe technique that produces a super-resolution (<20 nm) 3D topographic image of the surface of living cells (34, 67). SICM was performed with high resistance (~100 MΩ) nanopipettes filled with solution containing 200 mM NaCl, 4 mM CsCl, 1 mM CaCl_2_, 2 mM MgCl_2_, 0.05 mM CdCl_2_, 10 mM HEPES, 0.2 mM NiCl_2_, 10 mM glucose, 0.03 mM niflumic acid, and 0.004 mM strophanthidin, and the pH was adjusted to 7.4 with CsOH, thus blocking K^+^ (with Cs^+^), Cl^–^ (with niflumic acid), and cation (with CdCl_2_) channels and the Na^+^ pump (with strophanthidin) and NCX (with NiCl_2_). Cardiomyocytes were bathed in solution containing 0.33 mM NaH_2_PO_4_, 5 mM HEPES, 1 mM CaCl_2_, 10 mM EGTA, and 140 mM KCl, pH 7.4 with KOH, thus depolarizing the membrane potential to approximately 0 mV. Ionic currents were measured using an Axopatch 200B amplifier with Digidata 1440A (Molecular Devices) in voltage-clamp mode and monitored by the custom-modified universal controller (ICAPPIC Ltd.), which simultaneously controlled sample and pipette positioning. After visualizing the cell surface, the nanopipette was clipped in a controlled manner at a cell-free location until its resistance was reduced to approximately 20 MΩ. Next, a cell-attached patch-clamp recording was performed at a location chosen based on SICM topography. T-tubule openings were defined as deep niches located in z-grooves. After landing a pipette on a T-tubule opening, a gigaseal was formed by application of negative pressure. Only recordings obtained with 5 GΩ or higher seal resistance were used in this study. Currents were sampled at 10 kHz and filtered at 2 kHz. To assess the presence of fast inward currents, the membrane under the patch was held at 120 mV, and 1,000 ms voltage clamp pulses at 30 mV were applied every 3 seconds. Amplitudes of fast inward currents and late single-channel currents were assessed from 20–100 sweeps for the analyzed membrane patch. The number of Na_V_s in a membrane patch was calculated by dividing the maximal observed current amplitude by the average single-channel current amplitude measured during the late period (50–1,050 ms following the test potential application) (68). To measure burst activity in the late period, sweeps were idealized with a half-amplitude threshold–passing algorithm in Clampfit (version 10, Molecular Devices), with a threshold of 1.5 ± 0.1 pA (based on a Gaussian fit of histograms of recorded amplitudes). Quantification of burst activity was performed automatically using the custom Python script (available at https://github.com/tarasov4; branch name: Smart-patch-clamp-late-activity-analysis; commit ID: 2cbc19a) implementing density-based spatial clustering of applications with the noise (DBSCAN) algorithm (69). Since burst activity is defined as ion channel openings separated by brief closed periods (47), we analyzed burst activity as a function of closed periods within each sweep. Specifically, closed periods were plotted against their start and end times (Supplemental Figure 2). A cluster of at least 2 closed periods (corresponding to at least 3 openings) within a maximal distance between these of 2 ms was considered a burst opening. The total number of burst openings found in each membrane patch was then normalized to the total number of active channels per total number of sweeps.

### Fluorescence immunolabeling and confocal microscopy.

Immunofluorescence labeling of 2% PFA-fixed (MilliporeSigma) 5 μm tissue sections and cells (on coverslips) was performed as previously described (21, 35). Briefly, samples were permeabilized with Triton X-100 (0.2% in PBS, MilliporeSigma) for 15 minutes at room temperature and treated with a blocking agent (1% BSA, MilliporeSigma), followed by 0.1% Triton X-100 in PBS for 2 hours at room temperature prior to labeling with primary antibodies (overnight at 4°C). Proteins of interest were labeled with the following well-validated commercial or custom antibodies: ryanodine receptor 2 (RyR2) (mouse monoclonal antibody, catalog MA3-916, Thermo Fisher Scientific); NCX (mouse monoclonal antibody, catalog MA3-926, Thermo Fisher Scientific); Na_V_1.5 (a validated custom rabbit polyclonal antibody) (70); and Na_V_1.6 (a validated custom rabbit polyclonal antibody) (35). To ensure rigor, we further verified the selectivity of our Na_V_1.5 and Na_V_1.6 antibodies through additional negative controls (labeling with only primary or secondary antibodies, Supplemental Figure 3). FLAG-tagged CaM was labeled with an anti-DYKDDDDK–tagged antibody conjugated to Alexa Fluor 488 (catalog MA1-142-A488, Thermo Fisher Scientific). Samples were then washed in PBS (3 washes for 5 min each at room temperature) prior to labeling with secondary antibodies. For confocal microscopy, samples were then labeled with goat anti–rabbit Alexa Fluor 568 (1:4,000; Thermo Fisher Scientific); goat anti–mouse Alexa Fluor 488 (1:4,000; Thermo Fisher Scientific); and goat anti–mouse Alexa Fluor 647 (1:4,000; Thermo Fisher Scientific) secondary antibodies. Samples were then washed in PBS (3 washes for 5 min each at room temperature) and mounted in ProLong Gold (Thermo Fisher Scientific). For super-resolution STORM, samples were labeled with goat anti–mouse Alexa Fluor 647 (1:1,000) and goat anti–rabbit Biotium CF 568 (1:2,000) secondary antibodies (Thermo Fisher Scientific). Samples were then washed in PBS (3 washes for 5 min each at room temperature) and optically cleared using Scale U2 buffer (Thermo Fisher Scientific) for 48 hours at 4°C.

Confocal microscopy imaging was performed using a Nikon A1R-HD laser-scanning confocal microscope equipped with 4 solid-state lasers (405, 488, 560, and 640 nm, 30 mW each), a 63×/1.4NA oil-immersion objective, 2 GaAsP detectors, and 2 high-sensitivity photomultiplier tube detectors (Nikon). Individual fluorophores were imaged sequentially, with the excitation wavelength switching at the end of each line.

### Image analysis.

Spatial analysis of fluorescence images (RNA, protein) was performed using our previously published distance transform-based approach, morphological object localization (MOL) (71). This approach enables quantitative assessment of the localization of immunosignals relative to structural fiducials (cell periphery, nuclei) as well as to each other. Additionally, overlap between T-tubule–localized proteins (Na_V_1.5, Na_V_1.6, RyR2, and FLAG-tagged CaM) was assessed by examining the intensity profiles (generated using custom MATLAB code) from selected regions of interest.

### Further validation of genetic mouse lines.

We next applied confocal microscopy and MOL to verify normal localization of Na_V_1.5, Na_V_1.6, and RyR2, along with FLAG-tagged D96V-CaM in cD96V hearts, similar to the patterns observed in WT hearts (Supplemental Figures 4–6). To obtain further confirmation of the close association of Na_V_1.6 with RyR2 and FLAG-tagged CaM along T-tubules, we performed immunolabeling experiments in detubulated myocytes. Detubulation was achieved with a 15-minute incubation of freshly isolated cardiac myocytes in Tyrode’s solution with 1.5 M formamide (MilliporeSigma) as previously described (72) (Supplemental Figures 7 and 8). Whereas detubulation disrupted the close association between Na_V_1.6 and RyR2 (Supplemental Figure 8C) and decreased their attraction for each other (Supplemental Figures 8B), the association of FLAG-tagged CaM with Na_V_1.6 (Supplemental Figure 8F) and RyR2 (Supplemental Figure 8I) remained intact even after the T-tubules were disrupted. Notably, our Na_V_1.6 antibody displayed no observable immunoreactivity in cD96V cNa_V_1.6-KO hearts (Supplemental Figure 4), further demonstrating the antibody’s specificity.

### STORM super-resolution imaging.

STORM imaging was performed as previously described (33, 35). Briefly, imaging was performed using a Vutara 352 microscope (Bruker Nano Surfaces) equipped with biplane 3D detection and a fast scientific complementary metal-oxide semiconductor (sCMOS) camera, achieving 20 nm lateral and less than 50 nm axial resolution. Individual fluorophore molecules were localized with a precision of 10 nm. Registration of the 2 color channels was accomplished using localized positions of several TetraSpeck Fluorescent Microspheres (Thermo Fisher Scientific) scattered throughout the field of view. Protein distributions and organization were quantitatively analyzed using STORM-RLA, as previously described (33).

### Confocal Ca^2+^ imaging of intact cardiomyocytes.

Ventricular myocytes were loaded with 8 μM Fluo-4 AM (Invitrogen, Thermo Fisher Scientific) for 25 minutes at room temperature, followed by 25 minutes of incubation in fresh external solution (deesterification) containing 140 mM NaCl, 5.4 mM KCl, 1.0 mM CaCl_2_, 0.5 mM MgCl_2_, 10 mM HEPES, and 5.6 mM glucose (pH 7.4, NaOH). Intracellular Ca^2+^ cycling was assessed using a Nikon A1R-HD laser-scanning confocal microscope with 488 nm excitation and 500–600 nm light collection. Myocytes were paced at 0.3 Hz using extracellular platinum electrodes. Only cells not exhibiting spontaneous Ca^2+^ oscillations and showing full recovery of Ca^2+^ transients in response to an electric stimuli were analyzed to obtain the Ca^2+^ wave frequency. Ca^2+^ sparks were recorded between stimuli and analyzed using Spark Master. To assess the SR Ca^2+^ load, 20 mM caffeine was applied at the end of the experiments. All experiments were performed at room temperature (26°C).

### In vivo surface ECGs.

Continuous ECG recordings (PL3504 PowerLab 4/35, ADInstruments) were obtained from mice anesthetized with isoflurane (1%–3% isoflurane plus pure oxygen, 1 L/min). After 5 minutes of baseline recording, an i.p. injection of carbachol (0.5 mg/kg, MilliporeSigma) to simulate bradycardic conditions was administered, and the recording continued for an additional 20 minutes. Only animals that achieved heart rates below 200 beats/minute with carbachol were included in the analysis. ECG recordings were analyzed using the LabChart 7.3 program (ADInstruments). VT was defined as 3 or more consecutive premature beats (21). QT intervals were corrected for heart rate (QT_c_) (8) as follows: 

 (Equation 1) 
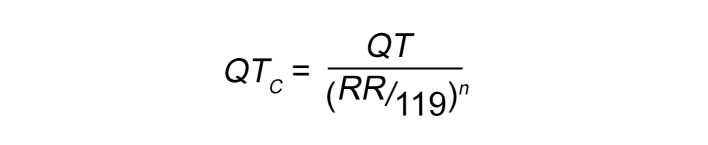


where QT and RR are the durations of the corresponding intervals (ms), 119 is the mean RR duration (ms) among all mice included in the analysis, and *n* is the slope factor in the linear regression equation: 

 (Equation 2) 
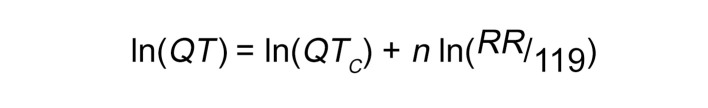


(Supplemental Figure 9).

### Statistics.

Statistical analyses were performed with GraphPad Prism 9 (GraphPad Software). The normality of the data was tested (Shapiro-Wilk test), and appropriate methods were chosen for comparative statistics. For comparison of 2 independent data sets, an unpaired, 2-tailed Student’s *t* or Mann-Whitney *U* test was used for normally and non-normally distributed data, respectively. For comparison of 2 paired data sets, the Wilcoxon matched-pairs, signed-rank test was used. A *P* value of less than 0.05 was considered significant. For comparison between >2 datasets, ordinary 1-way ANOVA or Kruskal-Wallis test were used for normally and non-normally distributed data, respectively. Post hoc multiple comparisons were performed with the original FDR method of Benjamini and Hochberg. A *q* value of less than 0.05 was considered significant. The χ^2^ and Fisher’s exact tests were used to compare categorical data. A *P* value less than 0.05 was considered significant. All data are expressed as mean ± SEM or as box and whiskers plots, where the box represents the first and third quartiles, the line within the box reflects the sample median, and the whiskers reflect the minimum and the maximum values, unless otherwise indicated. The *n* denotes the number of cells and *N* the number of mice.

### Study approval.

All animal procedures were approved by IACUC of The Ohio State University and performed in accordance with the NIH’s *Guide for the Care and Use of Laboratory Animals* (National Academies Press, NIH Publication No. 85-23, revised 2011).

## Author contributions

RV and PBR conceived and designed the study. MT, YO, HLS, AM, VB, RT, AMS, MD, DM, GS, ID, XM, and ADD performed experiments. JPD, DT, RV, and SG contributed tools. MT, YO, HLS, AM, RT, AMS, ADD, MPF, RV, and PBR analyzed data. MT, HLS, RV, and PBR drafted the manuscript or revised it critically for important intellectual content.

## Supplementary Material

Supplemental data

## Version 1 02/23/2023

In-Press Preview

## Version 2 04/03/2023

Electronic publication
