# Supplementary material for: NaV1.6 dysregulation within myocardial T-tubules by D96V calmodulin enhances proarrhythmic sodium and calcium mishandling
Source: J Clin Invest. 2023 Apr 3;133(7):e152071. doi: 10.1172/JCI152071 (PMC10065082; doi:10.1172/JCI152071)
Supplement: Supplemental data [file jci-133-152071-s039.pdf]

SUPPLEMENTAL MATERIALS, METHODS AND RESULTS

RNAscope

In situ hybridization was performed as previously described (1). Fresh frozen tissue sections were attached on slides then were fixed in 4% PFA in 1x PBS + 0.1% Diethyl Pyrocarbonate (DEPC, Sigma-Aldrich, Bornem, Belgium) for 15 min at 4°C, then rinsed with 1x PBS + 0.1% DEPC, followed by serial dehydration in 50%, 70% and 100% ethanol (Sigma-Aldrich, Bornem, Belgium) for 5 min each. Finally, samples were transferred to fresh 100% ethanol and stored at -20°C for up to two weeks. Tissue sections, thus stored, were air dried for 5 min then hydrophobic barrier were drawn and 80μM RNAscope® Hydrogen Peroxide (Advanced Cell Diagnostics Inc., Newark, CA, USA) solution was added as the barrier get completely dried. Slides were incubated for 10 min at RT then washed in 1XPBS+0.1%DEPC twice. Then 80μM of protease IV (RNAscope®) was added to each sample, slides were incubated for 30 min at RT and washed in 1XPBS+0.1%DEPC twice. Hybridization and incubation steps were performed in a humidified, covered chamber. For each incubation step, were used 80μl solution per one barriered section.

Individual mRNA molecules were visualized using RNAscope® Multiplex Fluorescent Reagent Kit v2 (Advanced Cell Diagnostics Inc., Newark, CA, USA), implemented per manufacturer-recommended protocols. Confocal microscopy was performed as previously described (2). Briefly, fluorescent signals were acquired using an A1R-HD laser scanning confocal microscope equipped with four solid-state lasers (405 nm, 488 nm, 560 nm, 640 nm, 30 mW each), a 60x/1.4 numerical aperture oil immersion objective, two GaAsP detectors, and two high sensitivity photomultiplier tube detectors (Nikon, Melville, NY, USA). At multicolor data acquisition, individual fluorophores were imaged sequentially with the excitation wavelength switching at the end of each frame. Probes were hybridized to the mRNA species of interest: *Scn5a* ($Na_V1.5$) (Cat No. 429881, NM_021544.4), *Scn8a* ($Na_V1.6$; Cat No. 434191, NM_001077499.2), following which cells were incubated in a series of amplification reagents provided by RNAscope®, before finally being labeled with one of the following fluorophores Opal 520 (FP1487001KT), Opal 570 (FP1488001KT) or Opal 690 (FP1497001KT) (all from Akoya Biosciences, Marlborough, MA, USA). Opal fluorophores were diluted 1:750.

Western blot

To assess $Na_V1.5$, $Na_V1.6$ and CaM expression in mouse myocardium tissue hearts were rapidly excised from isoflurane-anesthetized mice, were homogenized using Tissue Tearor (BioSpec Products, Inc., Bartlesville, OK, USA) in lysis buffer (Cell Signaling Technology, Danvers, MA, USA, Cat# 9803S) , supplemented with phosphatase (Calbiochem, Cat#524,625) and protease inhibitor cocktails (Sigma, Cat#P8340) as described previously (3). Samples were probed using custom previously validated rabbit polyclonal anti-$Na_V1.5$ (4), anti-$Na_V1.6$ (5) antibodies, rabbit polyclonal anti-CaM antibody (Cell Signaling Technology, Danvers, MA, USA, Cat# 4830), mouse monoclonal anti-GAPDH antibody (Abcam, Cambridge, UK, Cat# ab8245) followed by secondary anti-rabbit or anti-mouse IgG (H+L), HRP conjugated antibodies (Promega, Madison, WI, USA, Cat# W4011 and W4021, respectively). Blots were developed with ECL (Bio-Rad Laboratories) and quantified using Image J (US National Institutes of Health) and Origin 8 software (OriginLab Corporation, Northampton, MA, USA).

Fibrosis assessment

Masson`s trichrome tissue sectioning and staining were performed by the Comparative Pathology and Digital Imaging Lab on the Ohio State University veterinary campus. Briefly, fibrosis was assessed via Masson's trichrome staining from cryosections (5 μm thickness) of mouse hearts. Stained sections were imaged in entirety by tile-scanning with a 20X objective on a wide field

microscope (EVOS imaging system, ThermoFisher Scientific, Grand Island, NY, USA)) and the images analyzed using the automated fibrosis analysis toolkit as previously described (6).

Cardiac functional MRI

MRI imaging was performed by the Small Animal Imaging Core at OSU. Briefly, MRI images were obtained using 9.4T Bruker BioSpec system (Bruker, Ettlingen, Germany) equipped with 40 mm 1H quadrature volume resonator, and ParaVision 6.0.1 software. Animals were maintain under anesthesia during the acquisition with 1 - 1.5% of isoflurane mixed with carbogen (1L/min, 95%O2 and 5% CO2). Physiological parameters were monitored using Small Animal Monitoring and Gating System (SAI Inc., Stony Brook, NY). After a localizer image and FLASH-cine (Fast low angle shot) image with 4 chamber view, FLASH-cine images were acquired with following parameters: echo time TE=2.3 ms, repetition time TR=8 ms, flip angle FA=18°, number of averages NA=6, field of view 30 ×30 mm^2, matrix 230 × 230, slice thickness 1.0 mm. Multiple slices were acquired to cover entire left ventricle. All images were acquired with ECG and respiratory gating.

Epi- and endocardial surface of left ventricle were manual traced for each slice and used to obtain following functional parameters: end-diastolic (ED) and end-systolic (ES) volume, ED and ES mass, Stroke Volume (SV), cardiac output (CO), and Ejection Fraction (EF). ImageJ (https://imagej.nih.gov/ij/index.html) and ITK-SNAP (7) software have been used for creating stacks and mask for data analysis.

Transient transfection of mouse Na_V1.5 and Na_V1.6 in Chinese hamster ovary cells

Mouse *Scn8a* (accession no.: NM_001077499.2, encodes mNa_V1.6,) and mouse *Scn5a* (accession no.: NM_021544.4, encodes mNa_V1.5) coding sequences inserted into pcDNA3.1(+) P2A-eGFP were generated by GeneScript (Piscataway, NJ, USA). Chinese hamster ovary (CHO) - K1 cells (ATCC, Manassas, VA, USA) were transfected in 24-well plates with 500 ng of DNA per well using Lipofectamine 3000 (ThermoFisher Scientific, Grand Island, NY, USA) according to the manufacturer protocol. Cells were used 48 - 96 hours post transfections.

Dose-response of the 4,9ahTTX blocking effect on human and mouse Na_V1.5 and Na_V1.6

Human Na_V1.5 (hNa_V1.5) or human Na_V1.6 (hNa_V1.6) were stably expressed in Chinese hamster ovary (CHO) cells (B'SYS GmbH, Witterswil, Switzerland). Mouse Na_V1.5 (mNa_V1.5) or mouse Na_V1.6 (mNa_V1.6) were transiently expressed in CHO-K1 cells (ATCC, Manassas, VA, USA) and GFP positive cells 48 - 96 hours post transfection were used for experiments. Recordings of peak I_{Na} of human and mouse Na_V isoforms were performed under identical conditions. Specifically, cells were bathed in the solution containing (mM) 140 NaCl, 4 CsCl, 1 $CaCl_2$, 2 $MgCl_2$, 0.05 $CdCl_2$, 10 HEPES, 10 glucose, 0.03 niflumic acid, 0.004 strophanthidin, pH 7.2 (adjusted with CsOH). I_{Na} was measured in the whole cell path clamp configuration with the pipette solution containing (in mM) 10 NaCl, 20 TEACl, 123 CsCl, 1 $MgCl_2$, 0.1 Tris-GTP, 5 MgATP, 10 HEPES, and 10 EGTA to maintain free Ca^{2+} at ~0 nM, pH 7.2 (adjusted with CsOH). After establishment of the whole cell configuration cells were equilibrated for 5 minutes before the start of the experiment. The effects of 4,9ahTTX were assayed during measurements of a time course of peak I_{Na}. To measure a time course of peak I_{Na} cells were held at -120 mV and depolarization pulses to -30 mV (for hNa_V1.5 and mNa_V1.5) or to -10 mV (for hNa_V1.6 and mNa_V1.6) for 50 ms every 10 seconds were applied to elicit I_{Na}. In the beginning of the experiments, stability of peak I_{Na} amplitudes was checked during at least 10 depolarization pulses and the mean peak I_{Na} was established as a baseline peak I_{Na}. 4,9ahTTX was washed in the bath solution to achieve the desired final concentration. A response of peak I_{Na} was measured as the mean of at least 10 I_{Na} peaks after 10 minutes of bathing a cell at the fixed 4,9ahTTX concentration. Next, the bath was extensively perfused with the control bath solution during 10 – 15 minutes to wash out 4,9ahTTX and returning

of the peak I_{Na} amplitude to the baseline level was checked. The blocking effect of 4,9ahTTX was calculated as a fraction (%) of reduction of peak I_{Na} under 4,9ahTTX relative to baseline peak I_{Na}. Dose-response curves were fitted to the specific binding with Hill slope model (8).

Isothermal titration calorimetry (ITC) at 10 µM Ca^{2+}

Experiments were performed on a Microcal VP-ITC (Malvern Instruments, Malvern, UK) at 25°C in buffer containing 10 mM Mops, 2 mM EGTA, 1 mM TCEP, 150 mM KCl, and 1.93 mM $CaCl_2$ pH 7. Concentration of free Ca^{2+} in the EGTA – Ca^{2+} buffer solution was calculated with MaxChelator (9) and equal 10 µM. Na_V1.6CTD (73 - 110 µM) peptide was titrated with WT-CaM or D96V-CaM (12 µM and 16 µM, respectively). Concentration of free Ca^{2+} in the EGTA – Ca^{2+} buffer solution after adding 12 µM of apoCaM was calculated using the experimentally validated Ca^{2+}-CaM binding model (10) and was equal 9.7 µM. Titrations were performed with 28 injections, 1 of 5 µL and 27 of 10 µL, with 4 minute spacing between injections. Raw thermograms were processed using NITPIC (11), isotherms were fit using SEDPHAT (12), and visualized using GUSSI (13) (all programs from The University of Texas Southwestern Medical Center, Dallas, TX, USA).

Whole-cell patch clamp recordings of I_{Ca}

Ca^{2+} currents (I_{Ca}) from mouse cardiomyocytes were recorded as previously described (14). Briefly, using pipette solution containing (in mM) 10 NaCl, 20 TEACl, 123 CsCl, 1 $MgCl_2$, 0.1 Tris-GTP, 5 MgATP, 10 HEPES, and 10 EGTA, pH 7.2 and Na^+-free bath bath solution: 140 TEA-Cl, 4 CsCl, 1 $CaCl_2$, 2 $MgCl_2$, 10 HEPES, 10 glucose, 0.03 niflumic acid, 0.004 strophanthidin, pH 7.4. I_{Ca} density was measured by holding cardiomyocytes at -80 mV and applying 500 ms depolarization steps from -60 to 40 mV in increment of 10 mV every 3.5 s. Peak I_{Ca} decay phase was fitted to the mono-exponential decay function.

REFERENCES

1. Bogdanov V et al. Distributed synthesis of sarcolemmal and sarcoplasmic reticulum membrane proteins in cardiac myocytes. *Basic Res Cardiol.* 2021;116(1):63.

2. Mezache L et al. Vascular endothelial growth factor promotes atrial arrhythmias by inducing acute intercalated disk remodeling. *Sci Rep.* 2020;10(1):20463.

3. Terentyev D et al. Hyperphosphorylation of RyRs underlies triggered activity in transgenic rabbit model of LQT2 syndrome. *Circ Res.* 2014;115(11):919–928.

4. Veeraraghavan R et al. The adhesion function of the sodium channel beta subunit (β1) contributes to cardiac action potential propagation. *Elife.* 2018;7. doi:10.7554/eLife.37610

5. Struckman HL et al. Super-Resolution Imaging Using a Novel High-Fidelity Antibody Reveals Close Association of the Neuronal Sodium Channel NaV1.6 with Ryanodine Receptors in Cardiac Muscle. *Microsc Microanal*. 2020;26(1):157-165.

6. Gratz D, Winkle AJ, Dalic A, Unudurthi SD, Hund TJ. Computational tools for automated histological image analysis and quantification in cardiac tissue. *MethodsX* 2020;7:22–34.

7. Yushkevich PA et al. User-guided 3D active contour segmentation of anatomical structures: significantly improved efficiency and reliability. *NeuroImage.* 2006;31(3):1116–1128.

8. Hargus NJ, Nigam A, Bertram EH 3rd, Patel MK. Evidence for a role of Nav1.6 in facilitating increases in neuronal hyperexcitability during epileptogenesis. *J Neurophysiol.* 2013;110(5):1144–1157.

9. Bers DM, Patton CW, Nuccitelli R. A practical guide to the preparation of Ca(2+) buffers. *Methods Cell Biol.* 2010;99:1–26.

10. Seeger C, Talibov VO, Danielson UH. Biophysical analysis of the dynamics of calmodulin interactions with neurogranin and Ca2+ /calmodulin-dependent kinase II. *J Mol Recognit*. 2017;30(8):e2621.

11. Keller S et al. High-precision isothermal titration calorimetry with automated peak-shape analysis. *Anal Chem.* 2012;84(11):5066–5073.

12. Zhao H, Piszczek G, Schuck P. SEDPHAT--a platform for global ITC analysis and global multi-method analysis of molecular interactions. *Methods*. 2015;76:137-148.

13. Brautigam CA. Calculations and Publication-Quality Illustrations for Analytical Ultracentrifugation Data. *Methods Enzymol.* 2015;562:109–133.

14. Wang X et al. Conditional knockout of Fgf13 in murine hearts increases arrhythmia susceptibility and reveals novel ion channel modulatory roles. *J Mol Cell Cardiol.* 2017;104:63–74.

SUPPLEMENTAL FIGURES

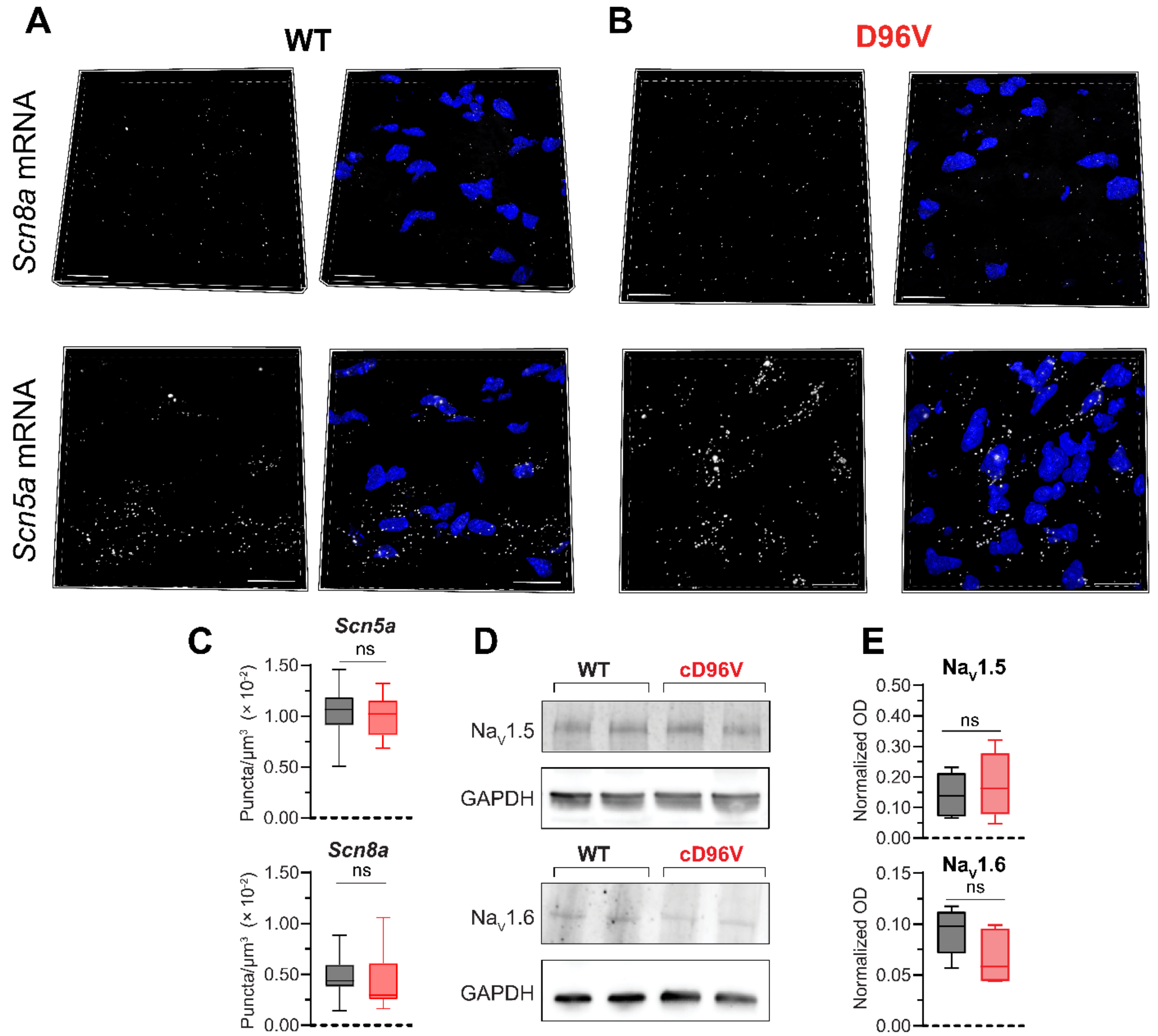

Figure S1. Expression of Na$_V$1.5 and Na$_V$1.6 in WT and cD96V hearts. Representative 3D confocal images showing *Scn8a* (encoding Na$_V$1.6) and *Scn5a* (encoding Na$_V$1.5) mRNA (white dots; RNAScope^{TM} *in situ* hybridization as previously described (1)) in **A)** WT and **B)** cD96V myocardium. (Scalebars: 20 µm). **C)** Summary plot of mRNA puncta density. (n = 5 images/sample, N= 3 hearts/group (1 male, 2 females, 22 weeks old for WT and 1 male, 2 females, 21 – 31 weeks old for cD96V), Wilcoxon rank-sum test: non-significant (ns), $p > 0.05$) **D)** Representative Western immunoblots and **E)** summary data demonstrating comparable expression of Na$_V$1.6 and Na$_V$1.5 proteins in WT and cD96V hearts. OD – optical density. OD values of Na$_V$1.5 and Na$_V$1.6 blots were normalized to respective OD of GAPDH blots, N = 6 mice for Na$_V$1.5 and Na$_V$1.6 from WT (3 males, 3 females, 7 – 17 weeks old) and cD96V (3 males, 3 females, 7 – 17 weeks old). Non-significant (ns), $p > 0.05$ unpaired Student's t-test.

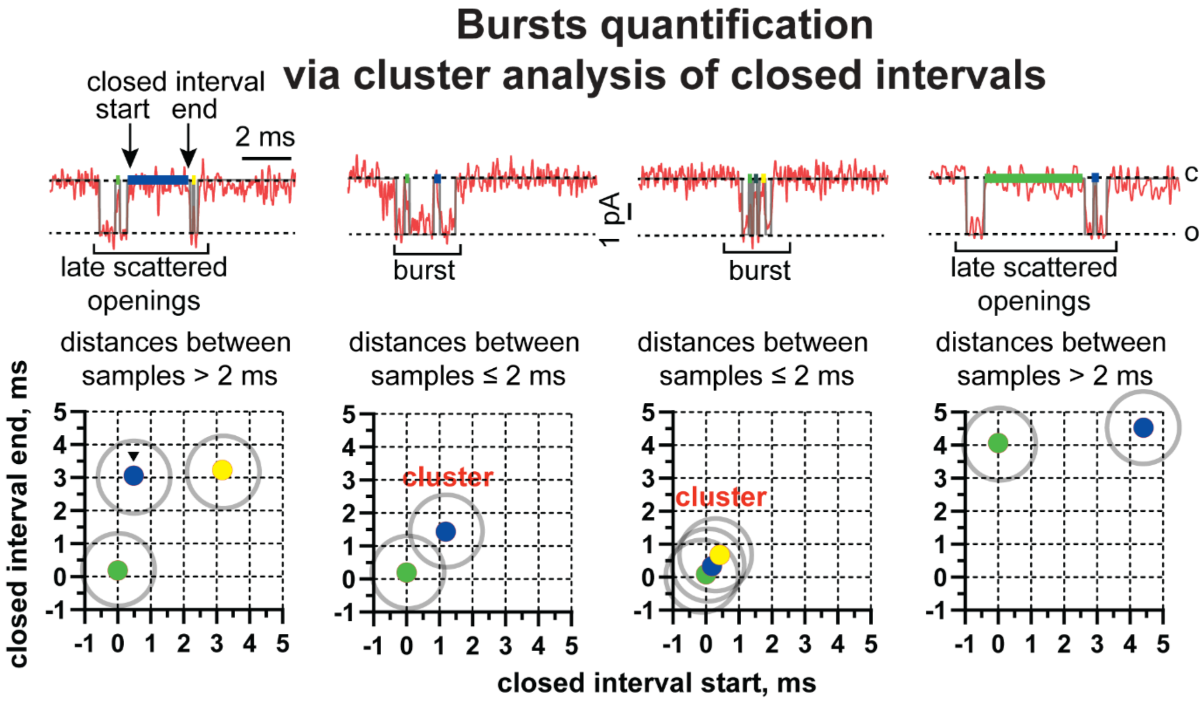

Figure S2. Burst activity quantification in “smart” patch clamp recordings. Upper panel: representative current sweeps obtained from T-tubules of cD96V cardiac myocytes. Red traces represent late activity within the late period (50 – 1050 ms following the test potential application). Sweeps were idealized with the half amplitude threshold passing algorithm (gray). Burst activity was analyzed as a function of closed periods within each sweep. Arrows in the first recording indicate start and end of one closed interval marked with the black triangle in the corresponding plot of closed intervals relative to their start and end times (colors of dots correspond to closed interval marked in the upper panel). Each closed period is encircled with a 2 ms diameter (maximal time distance between closed intervals to be included in one cluster of burst activity by DBSCAN algorithm). Only intervals with overlapped surrounding circles (panel 2 and 3) form clusters of burst activity.

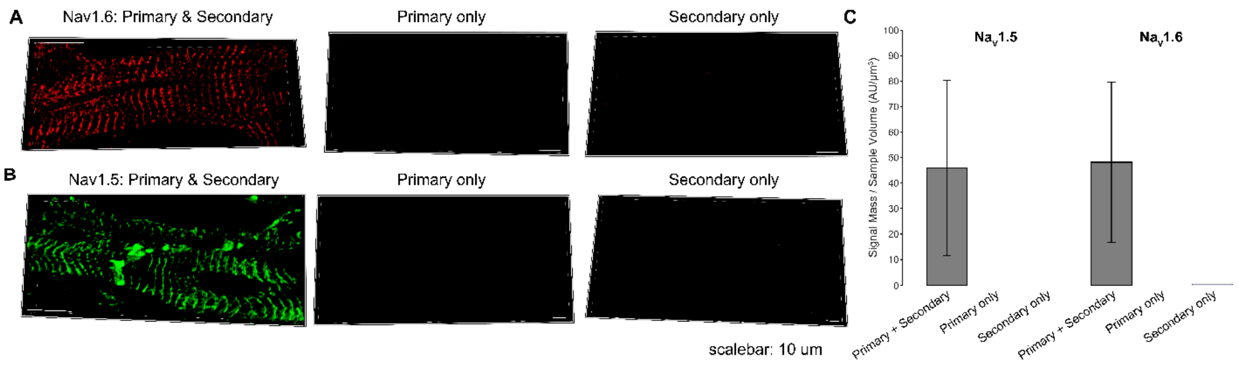

Figure S3. Validation of Na$_V$1.6 and Na$_V$1.5 antibodies. Representative 3D confocal images from positive (primary + secondary antibodies) and negative (primary or secondary antibody only) control experiments with **A)** Na$_V$1.6 (data obtained from a WT, female, 22 weeks old mouse) and **B)** Na$_V$1.5 (a WT, female, 22 weeks old mouse) antibodies (3 images/sample). Scale bars: 20 µm. **C)** Summary plot of immunofluorescent density.

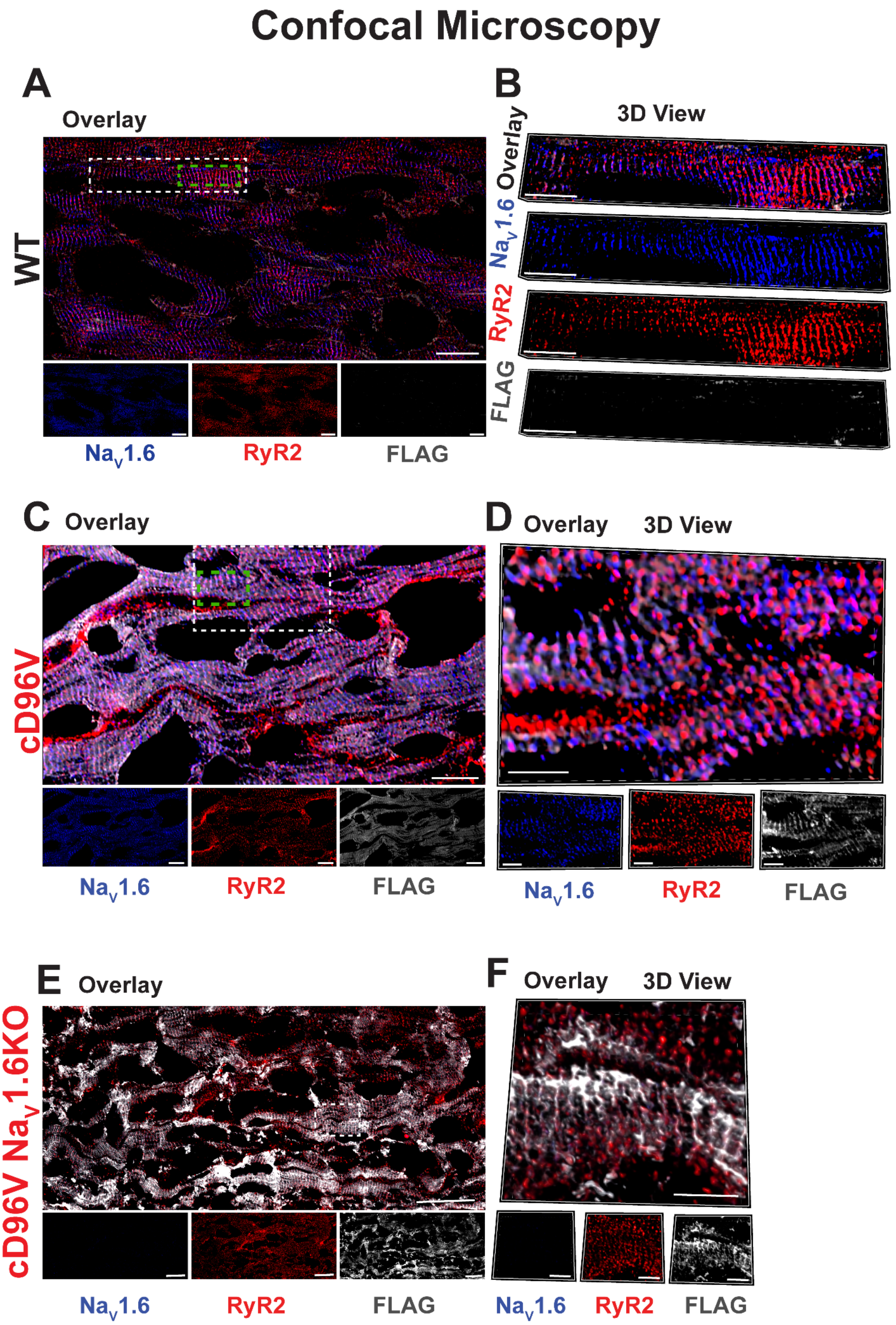

Confocal Microscopy
A
Overlay
WT
Na$_V$1.6
RyR2
FLAG
B
3D View
Overlay
Na$_V$1.6
RyR2
FLAG
C
Overlay
cD96V
Na$_V$1.6
RyR2
FLAG
D
Overlay
3D View
Na$_V$1.6
RyR2
FLAG
E
Overlay
cD96V Na$_V$1.6KO
Na$_V$1.6
RyR2
FLAG
F
Overlay
3D View
Na$_V$1.6
RyR2
FLAG

Figure S4. Na_V1.6 Localization. Representative confocal images showing distribution of Na_V1.6 relative to RyR2 and FLAG-tagged D96V-CaM in **A, B)** WT (female, 22 weeks old), **C, D)** cD96V (male, 21 weeks old) and **E, F)** cD96V x Na_V1.6KO (male, 7 weeks old) hearts. (Scale bars: A, C, E: 25 μm, B, D, F: 5 μm; dashed white boxes in A, C, E outline regions depicted in B, D, F respectively, dashed green boxes in A and C outline regions analyzed with FFT in Figure S6A-D).

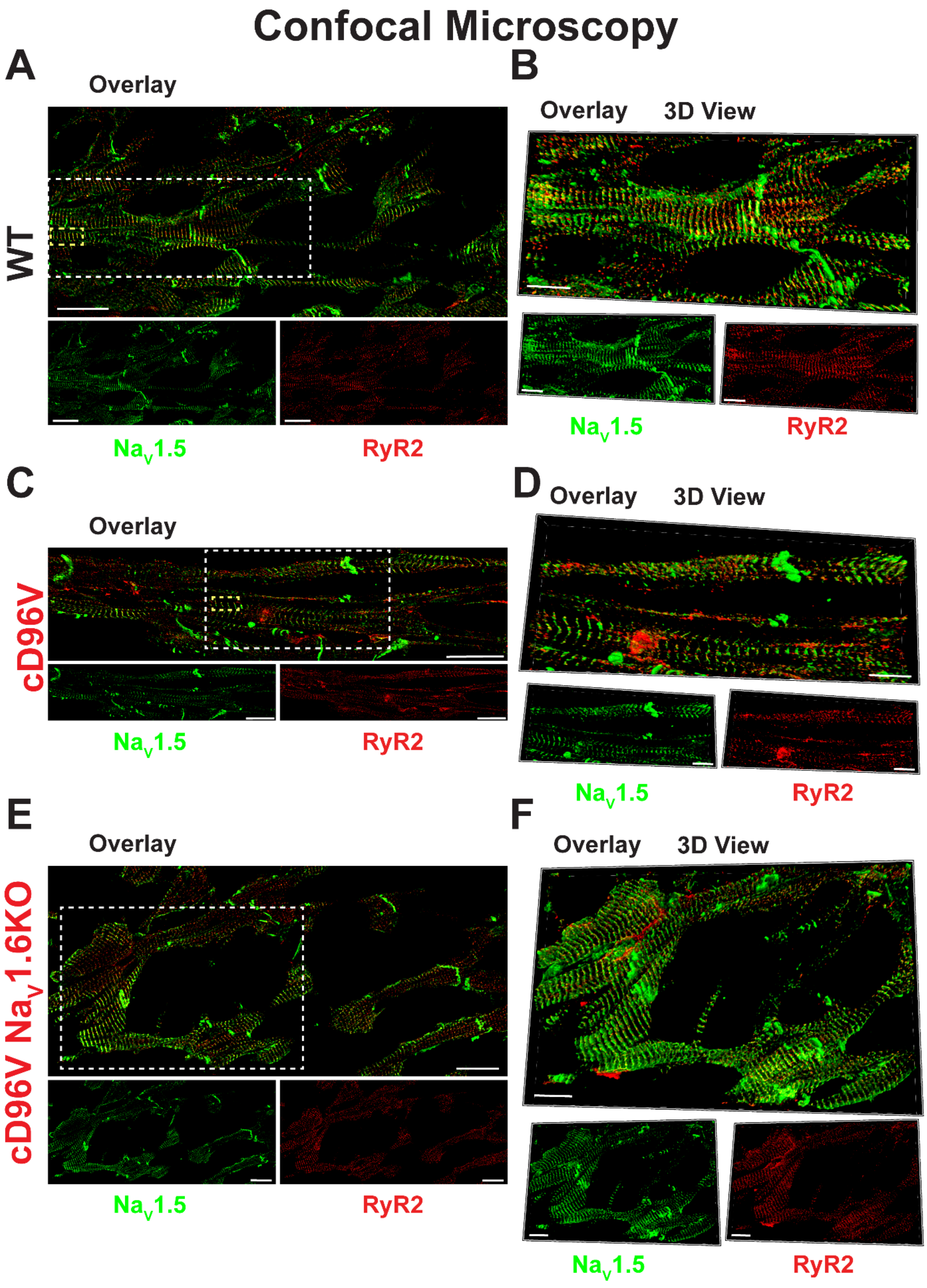
Confocal Microscopy
A
Overlay
WT
Nav1.5
RyR2
B
Overlay
3D View
Nav1.5
RyR2
C
Overlay
cD96V
Nav1.5
RyR2
D
Overlay
3D View
Nav1.5
RyR2
E
Overlay
cD96V Nav1.6KO
Nav1.5
RyR2
F
Overlay
3D View
Nav1.5
RyR2

190

Figure S5. $Na_V1.5$ Localization. Representative confocal images showing distribution of $Na_V1.5$ relative to RyR2 in **A, B)** WT (female, 22 weeks old), **C, D)** FLAG-tagged cD96V (male, 21 weeks old) and **E, F)** FLAG-tagged cD96V-$Na_V1.6$KO (male, 7 weeks old) hearts. (Scale bars: A, C, E: 25 µm, B, D, F: 10 µm; dashed white boxes in A, C, E outline regions depicted in B, D, F respectively, dashed yellow boxes in A and C outline regions analyzed with FFT in Figure S6F-I).

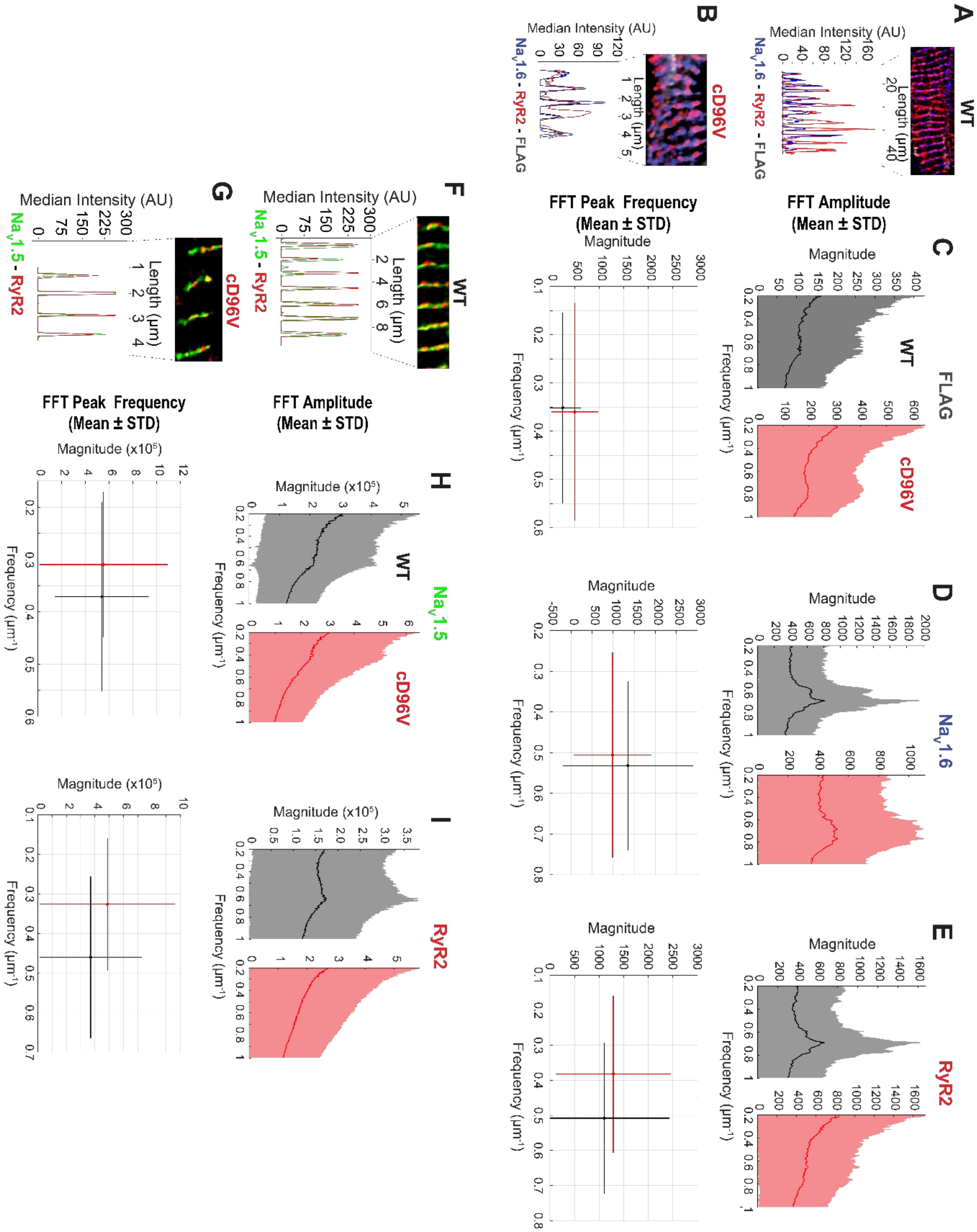

Figure S6. Fast Fourier transform (FFT) analysis of Na$_V$1.5, Na$_V$1.6, RyR2 and D96V-CaM co-localization. A, B) Fluorescence intensity profiles and **C-E)** Fourier transform analysis of Na$_V$1.6, RyR2 and D96V-CaM in WT and FLAG-tagged cD96V myocardium. A and B are crops of the images in Figure S4A and S4C highlighted by dashed green boxes. **F, G)** Fluorescence intensity profiles and **H, I)** Fourier transform analysis of Na$_V$1.5 and RyR2 in WT and cD96V myocardium. F and G are crops of the images in Figure S5A and S5C highlighted by dashed yellow boxes. (n = 3 images/sample).

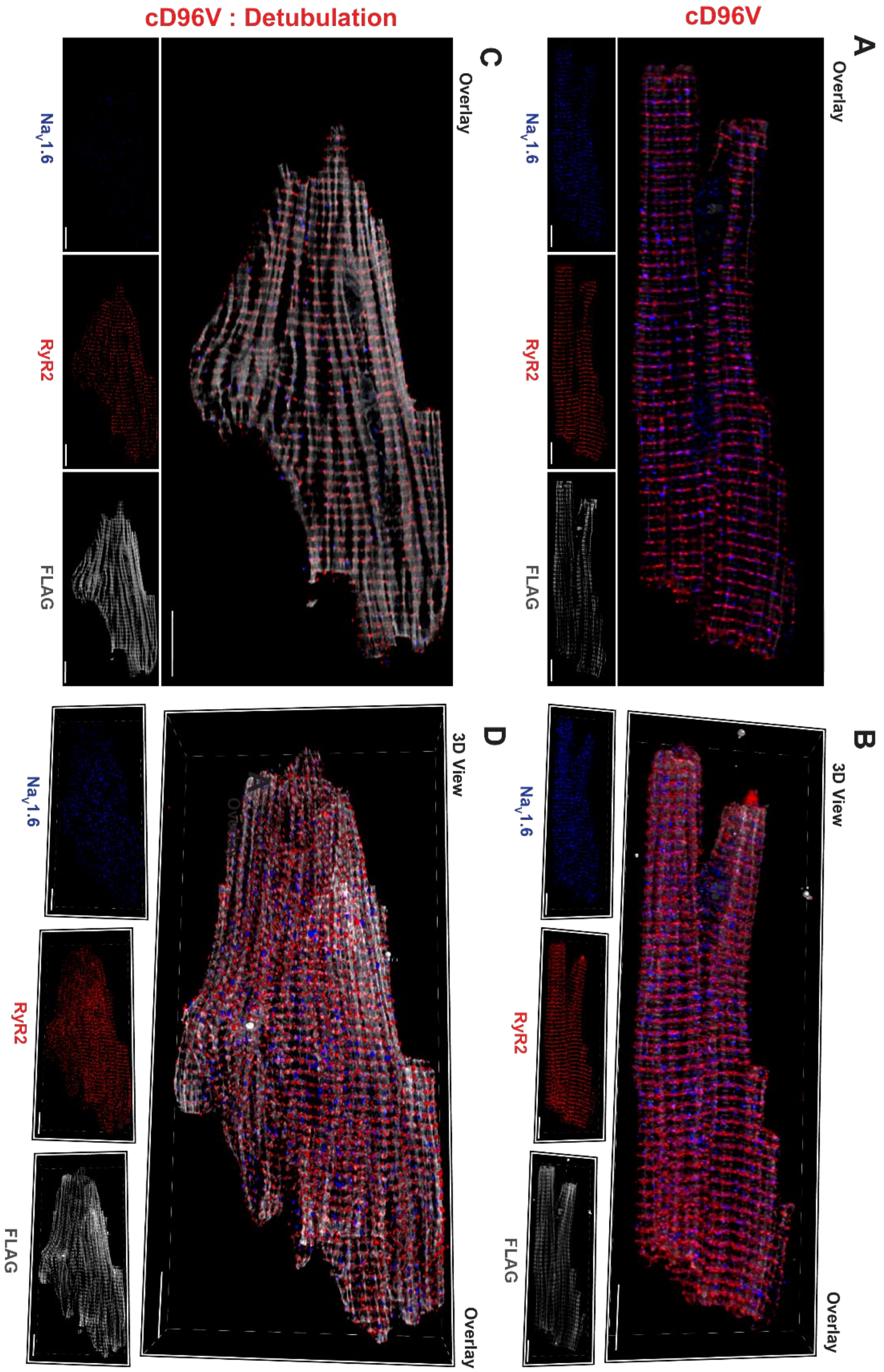

Figure S7. Effects of myocyte detubulation on Na_V1.6, RyR2 and D96V-CaM localization. Representative 2D and 3D confocal images of Na_V1.6, RyR2 and FLAG-tagged D96V CaM in FLAG-tagged cD96V myocytes (a male, 21 weeks old) subjected to **A, B)** vehicle treatment and **C, D)** detubulation by formamide (1.5 M).

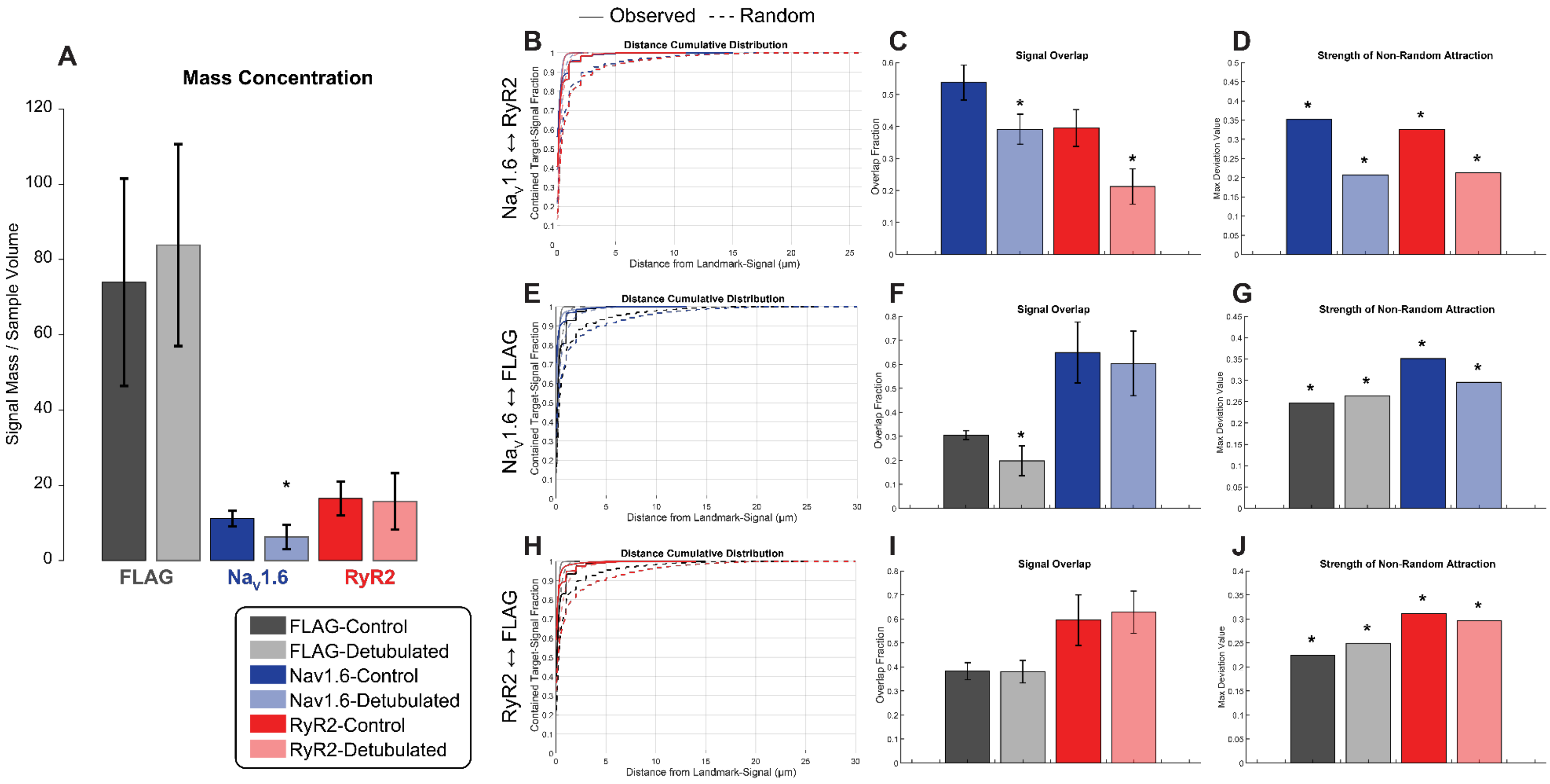
A
Mass Concentration
Signal Mass / Sample Volume
FLAG
NaV1.6
RyR2
*
FLAG-Control
FLAG-Detubulated
Nav1.6-Control
Nav1.6-Detubulated
RyR2-Control
RyR2-Detubulated
— Observed --- Random
B
NaV1.6 ↔ RyR2
Distance Cumulative Distribution
Contained Target-Signal Fraction
Distance from Landmark-Signal (µm)
C
Signal Overlap
Overlap Fraction
D
Strength of Non-Random Attraction
Max Deviation Value
E
NaV1.6 ↔ FLAG
Distance Cumulative Distribution
Contained Target-Signal Fraction
Distance from Landmark-Signal (µm)
F
Signal Overlap
Overlap Fraction
G
Strength of Non-Random Attraction
Max Deviation Value
H
RyR2 ↔ FLAG
Distance Cumulative Distribution
Contained Target-Signal Fraction
Distance from Landmark-Signal (µm)
I
Signal Overlap
Overlap Fraction
J
Strength of Non-Random Attraction
Max Deviation Value

Figure S8. Quantitative analysis of myocyte detubulation on Na_V1.6, RyR2 and D96V-CaM localization. A) Abundance of Na_V1.6, RyR2 and FLAG-D96V CaM immunosignal in control and detubulated FLAG-tagged cD96V myocytes. Summary results from Morphological Object Localization showing relative localization of **B-D)** Na_V1.6 vs. RyR2, **E-G)** Na_V1.6 vs. FLAG-D96V CaM, and **H-J)** RyR2 vs. FLAG-D96V. **B, E, H** show cumulative distributions for distances between immunosignals while **C, F, I** show degree of overlap. **D, G, J** show the degree of non-random attraction between immunosignals. (n= 8 cells/treatment and n = 7 cells/treatment for control and detubulation, respectively, *$p < 0.05$ Mann-Whitney test).

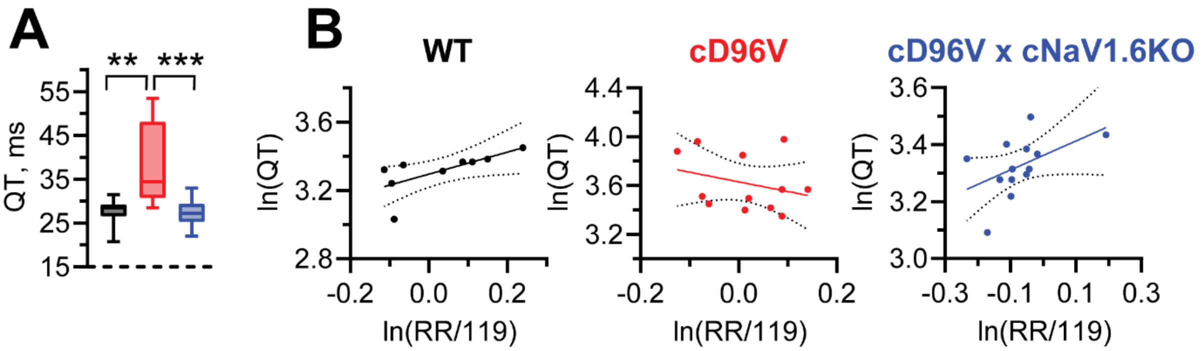

Figure S9. *In vivo* QT prolongation in cD96V. A) Summary data of baseline QT intervals from *in vivo* ECG measurements from WT (N = 9, 4 males, 5 females, 12 – 25 weeks old), FLAG-tagged cD96V (N = 12, 7 males, 5 females, 6 – 18 weeks old), FLAG-tagged cD96V× cNa$_V$1.6 (N = 13, 9 males, 4 females, 6 – 26 weeks old). Kruskal-Wallis test with original FDR method of Benjamini and Hochberg post hoc test, **q < 0.01, ***q < 0.001. **B)** Summary plots showing log-transformed QT intervals (ms) vs. RR intervals (normalized to the mean of RR intervals of all studied mice,119 ms). Experimental values are fit to the linear regression model: $\ln(QT) = \ln(QT_c) + n\ln({}^{RR}/_{119})$. Dashed lines indicate 95% confidence intervals.

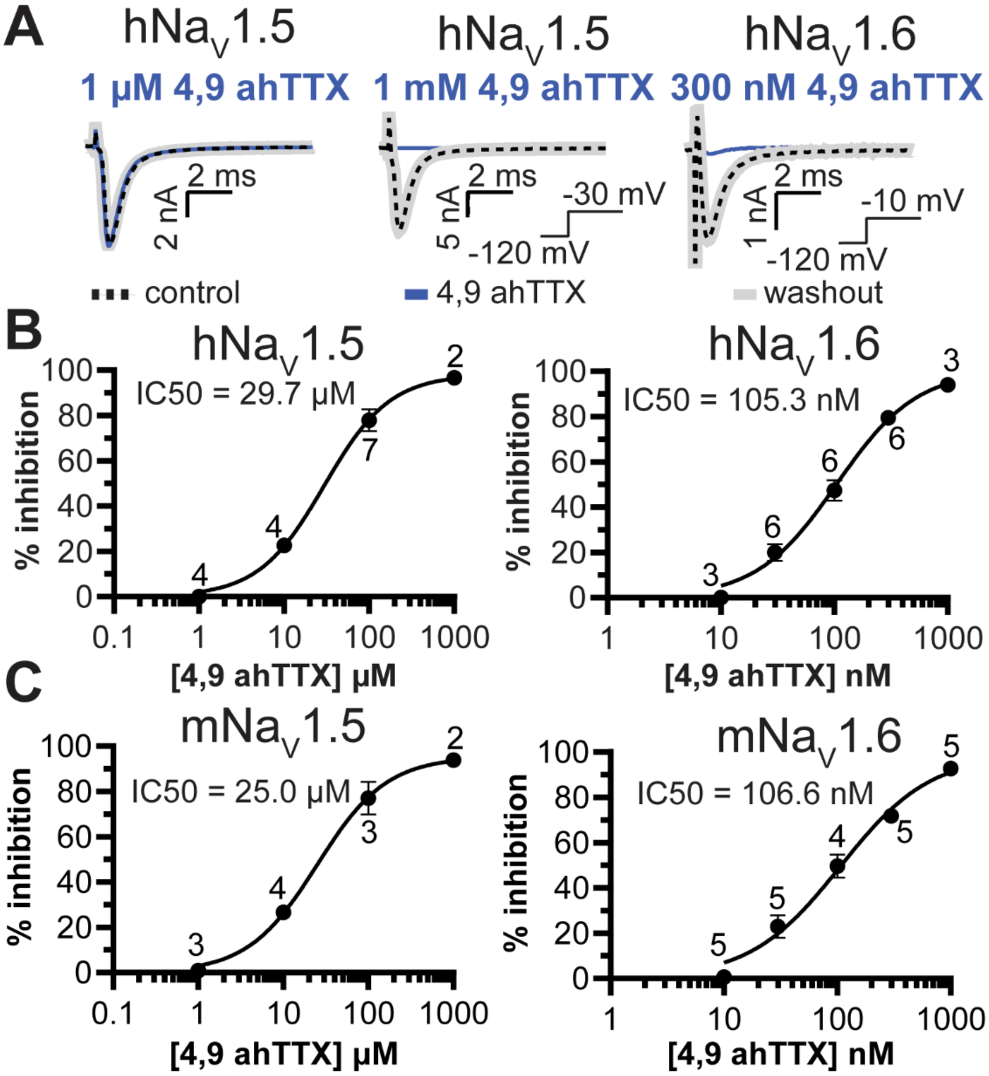

Figure S10. Selectivity of 4,9ahTTX for human and mouse Na$_V$1.6 blockade over Na$_V$1.5.

(A) Representative current traces recorded from CHO cells expressing human Na$_V$1.5 (hNa$_V$1.5) or Na$_V$1.6 (hNa$_V$1.6). Currents were recorded before application (black traces, control), 10 minutes after application (blue traces, concentrations of 4,9ahTTX shown in the figure) and 10 minutes after washout (gray traces) of 4,9ahTTX. Voltage protocols are shown in the figure. **(B)** Summary dose-response relationships for human (hNa$_V$1.5-, *left* and hNa$_V$1.6-, *right* stably expressing CHO cells), and **(C)** mouse Na$_V$s (mNa$_V$1.5, *left*, mNa$_V$1.6, *right*, transiently expressing CHO cells). Numbers of tested cells shown in the plots. Experimental values were fitted to specific binding with Hill slope model, half maximal inhibitory concentration (IC$_{50}$) values shown in the plots.

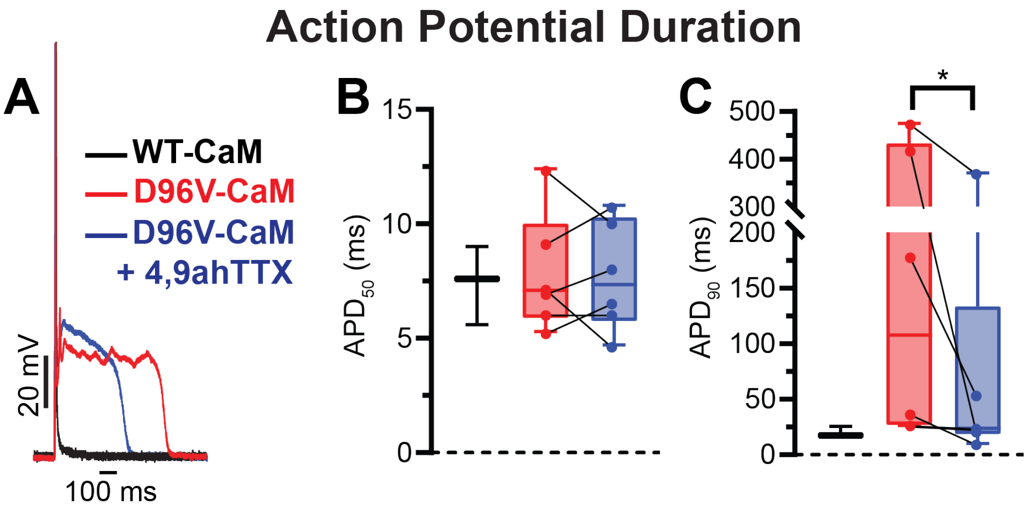

Figure S11. 4,9ahTTX ameliorates D96V-CaM-mediated action potential prolongation.

(A) Exemplar action potential (AP) traces recorded in mouse cardiomyocytes dialyzed with either WT-CaM (black), D96V-CaM (red) before or after application of 4,9ahTTX (300 nM; blue). In these experiments the recombinant CaMs were not FLAG-tagged. **(B)** APD$_{50}$ **(C)** and APD$_{90}$. For WT-CaM n = 3, N = 3 (3 males, 7 – 10 weeks old); D96V-CaM and D96V-CaM + 4.9ahTTX n = 6, N = 4 (3 males, 1 female, 14 – 17 weeks old). $*p < 0.05$ by Wilcoxon matched-pairs signed rank test.

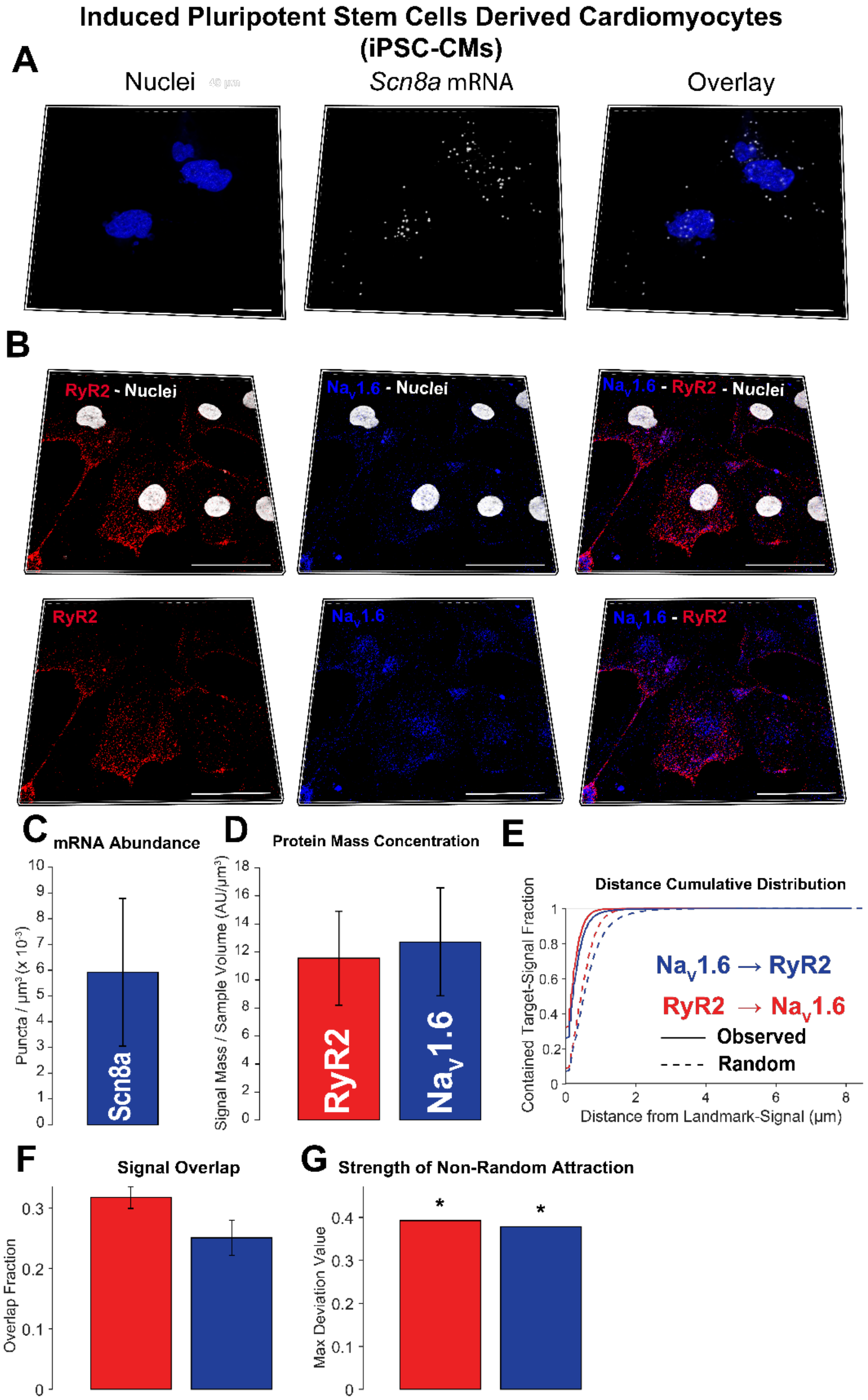

Figure S12. Expression of Na$_V$1.6 in human iPSC-CMs. Representative 3D confocal images showing **A)** *Scn8a* mRNA (scale bars: 40 μm) and **B)** Na$_V$1.6 protein (scale bars: 20 μm) in iPSC-CMs. Summary plots showing abundance of **C)** *Scn8a* mRNA abundance (n=5 images/sample) and **D)** Na$_V$1.6 protein (n=6 images/sample). **E)** Cumulative distribution of distances, **F)** degree of signal overlap and **G)** strength of non-random attraction between Na$_V$1.6 and RyR2. (* p < 0.05 Mann-Whitney test).

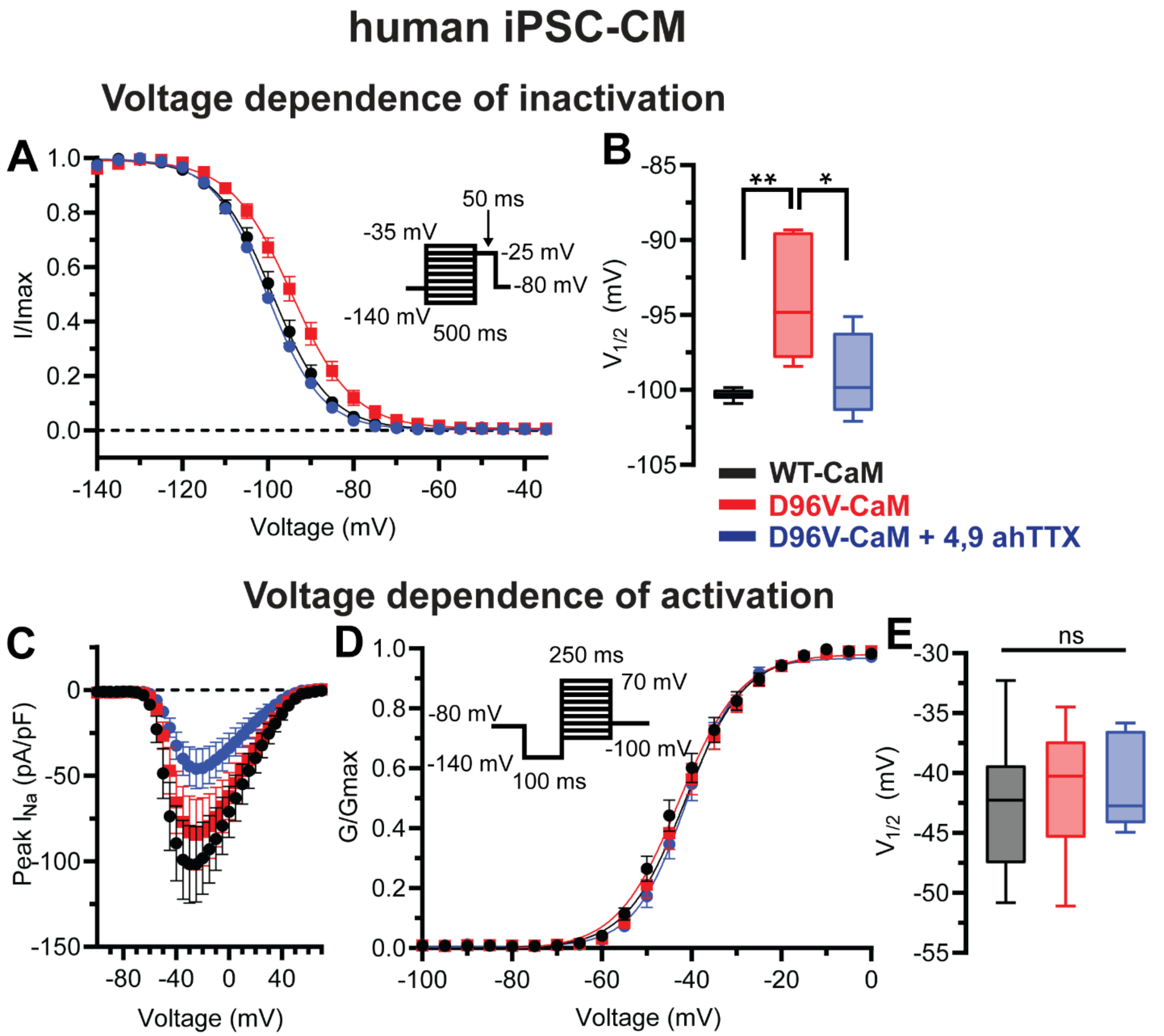

Figure S13. D96V-CaM shifts voltage dependence of I_{Na} inactivation in human iPSC-CMs. **(A)** Steady state inactivation curves **(B)** and corresponding $V_{1/2}$ of inactivation. For WT-CaM (black) n = 5; D96V-CaM (red) n = 7; D96V-CaM (blue) + 4.9ahTTX (300 nM) n = 5. **(C)** I-V relationship and **(D)** normalized I_{Na} conductance with **(E)** corresponding $V_{1/2}$ of activation. For WT-CaM n = 14; D96V-CaM n = 12; D96V-CaM + 4.9ahTTX (300 nM) n = 7. *$q < 0.05$ **$q < 0.01$, and $q > 0.05$ non-significant (ns) by ordinary one way ANOVA test with original FDR method of Benjamini and Hochberg for multiple comparison.

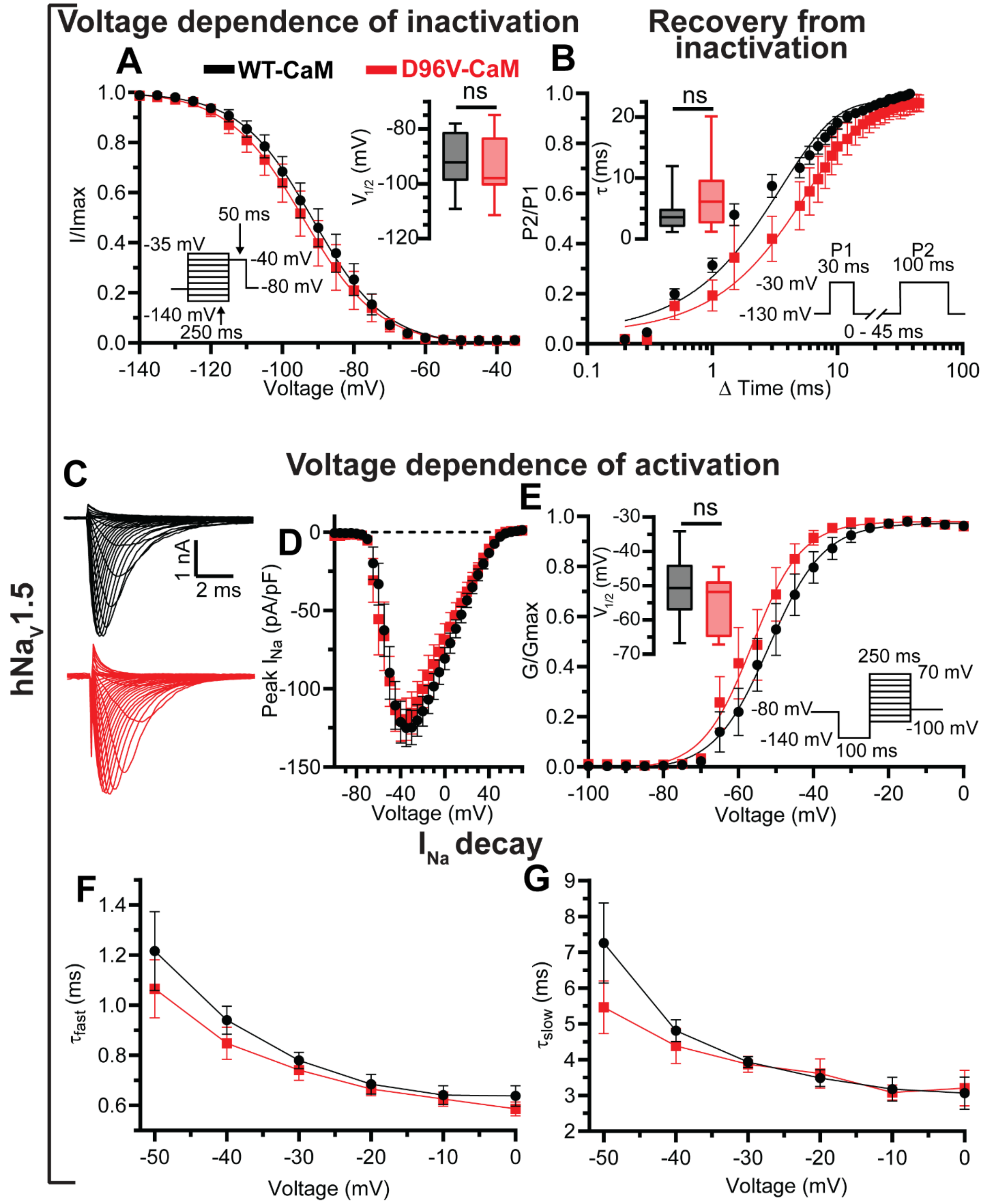

Figure S14. D96V-CaM does not affect Na$_V$1.5 function. (A) Voltage-dependence of steady state inactivation. Inset: $V_{1/2}$ of inactivation. For WT-CAM n = 12, D96V-CaM n = 10. In these experiments the recombinant CaMs were not FLAG-tagged. **(B)** Recovery from inactivation. Inset: Time constants (τ). For WT-CAM n = 14, D96V-CaM n = 8. **(C)** Whole-cell I_{Na}. Summary plots of

(D) I-V and **(E)** normalized conductance (G)-V relationships. Inset: $V_{1/2}$. For WT-CAM n = 12, D96V-CaM n = 8. **(F)** Fast (τ_{fast}) and **(G)** slow (τ_{slow}) decay time constants of peak I_{Na}. For WT-CaM n = 12, D96V-CaM n = 8. $p > 0.05$ by Student's t-test, except for fast inactivation (τ_{fast}) Mann-Whitney test.

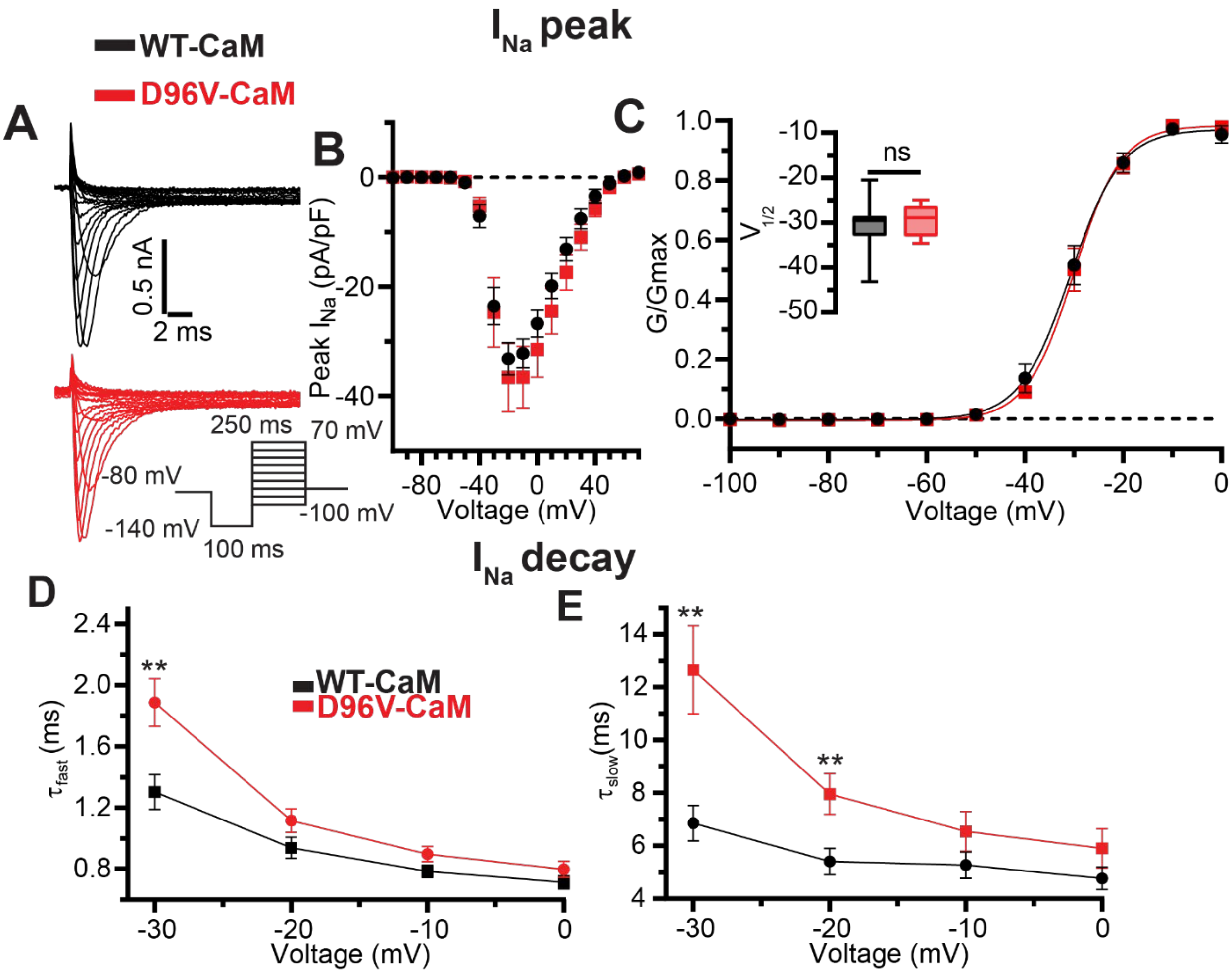

Figure S15. D96V-CaM effects on $Na_V1.6$ peak current. (A) Whole-cell peak currents, **(B)** I-V and **(C)** G-V relationships. Inset: $V_{1/2}$. $p > 0.05$ WT-CAM n = 12, D96V-CaM n = 8 Student's t-test. (**D**) τ_{fast} and (**E**) τ_{slow}. For WT-CaM and D96V-CaM n = 12 and n = 8, respectively; $^{**}p < 0.01$ Student's t-test. In these experiments the recombinant CaMs were not FLAG-tagged.

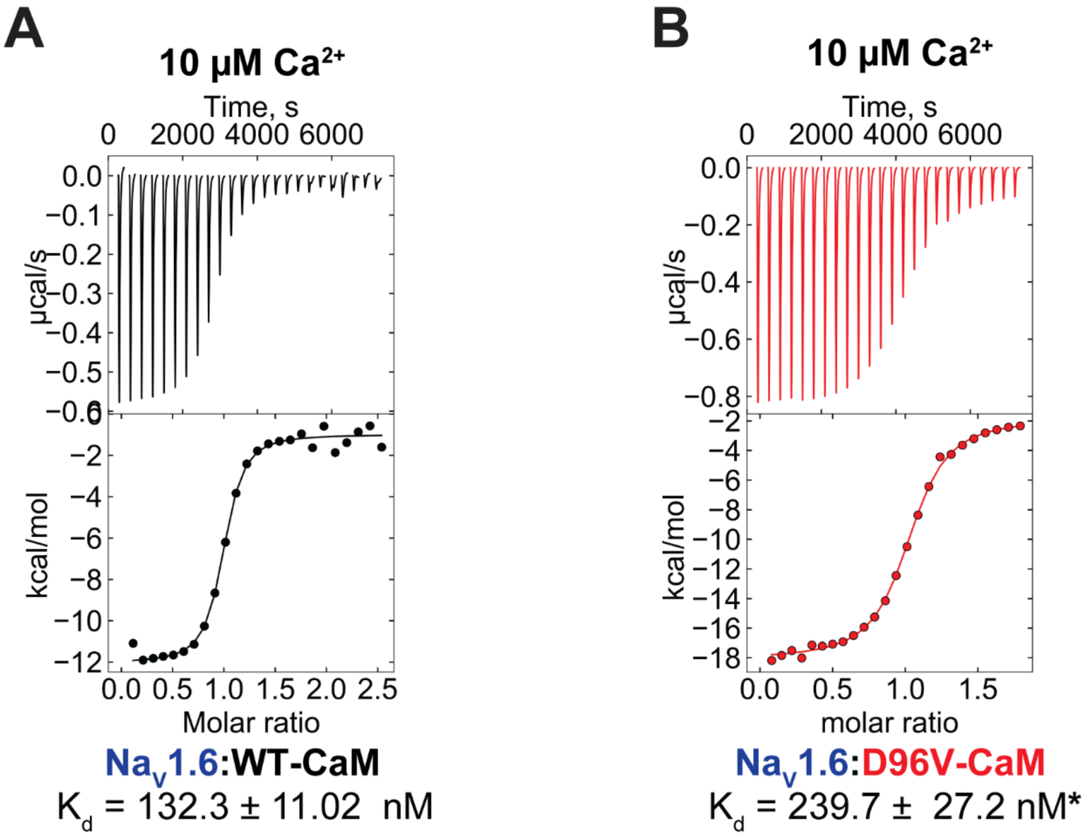

Figure S16. ITC measurements of CaM – Na_V1.6 interaction at 10 μM Ca^{2+}. (A) Representative ITC measurements of hNa_V1.6CTD (residues 1891–1918) binding to WT-CaM **(B)** and D96V-CaM at 10 μM of free Ca^{2+}. Raw (*top*) and cumulative (*bottom*) plots of heat evolved following injections. All curves are fitted to a one-binding site per monomer model. Three replicates for hNa_V1.6CTD:WT-CaM and hNa_V1.6CTD:D96V-CaM. For K_d of WT-CaM:hNa_V1.6 CTD vs. D96V-CaM:hNa_V1.6 CTD at 0 Ca^{2+} and 10 μM Ca^{2+} *$q < 0.05$ with one way ANOVA with Original FDR method of Benjamini and Hochber for post hoc comparison.

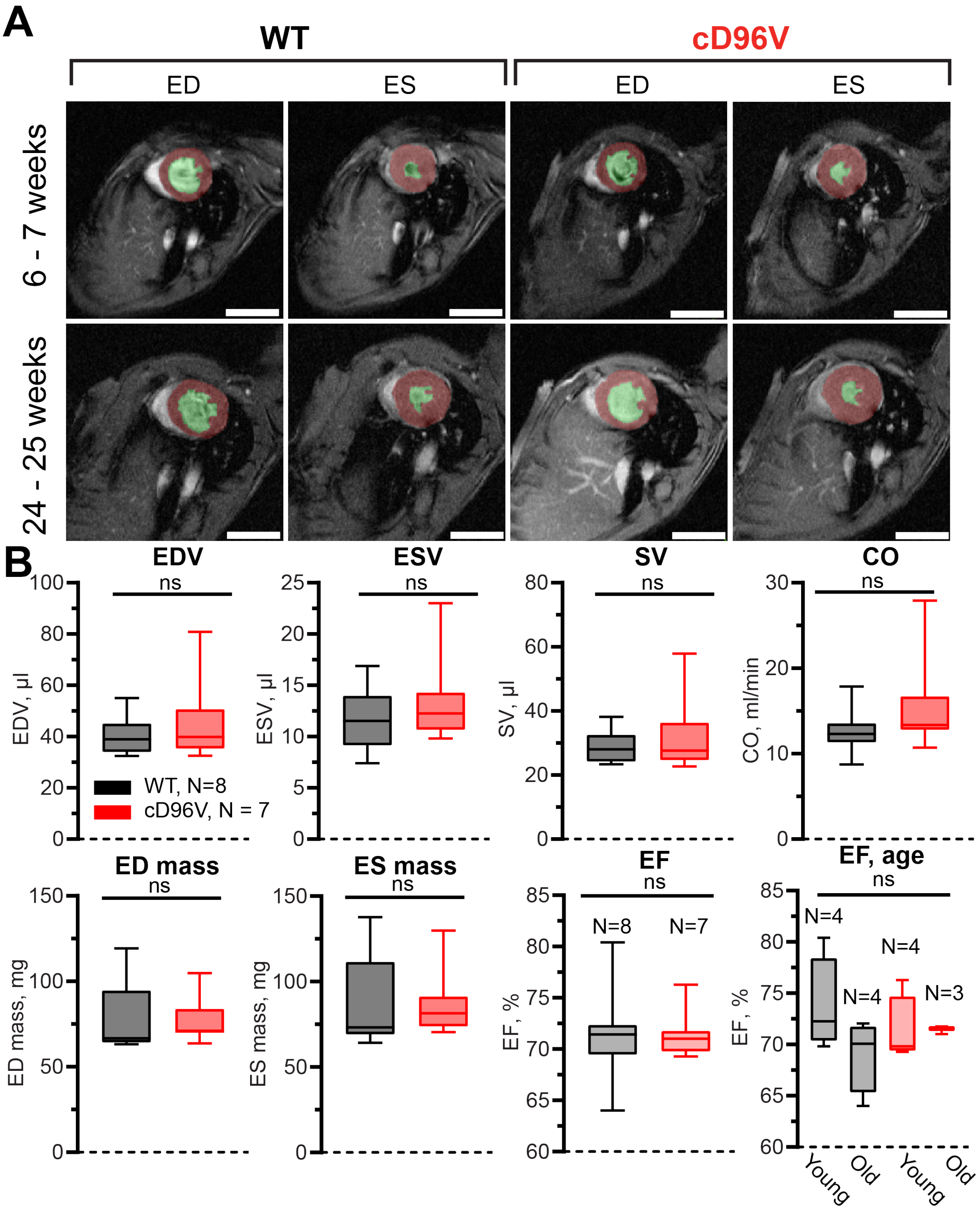

Figure S17. Magnetic resonance imaging (MRI) measurements of the cardiac mechanical function *in vivo*. (A) Representative fast low angle shot (FLASH) cine images of hearts at end diastole (ED) and end systole (ES) from young and old WT and FLAG-tagged cD96V mice with epicardial (brown) and endocardial (green) surfaces highlighted (Scale bars: 5 mm). **(B)** Summary

data: end-diastolic volume (EDV), end-systolic volume (ESV), stroke volume (SV), cardiac output (CO), end-diastolic mass (ED mass), end-systolic mass (ES mass), end ejection fraction (EF) for WT (N = 8, 3 males, 5 females, 6 – 24 weeks old) and cD96V (N = 7, 3 males, 4 females, 6 – 26 weeks old) mice. ns – non-significant, $p > 0.05$ Mann-Whitney test. EF was also separated by age (young: 6-7 weeks old, old: 24 – 25 weeks old, numbers of tested mice included in the figure). ns – non-significant, $p > 0.05$ Kruskal-Wallis test with post hoc original FDR method of Benjamini and Hochberg test.

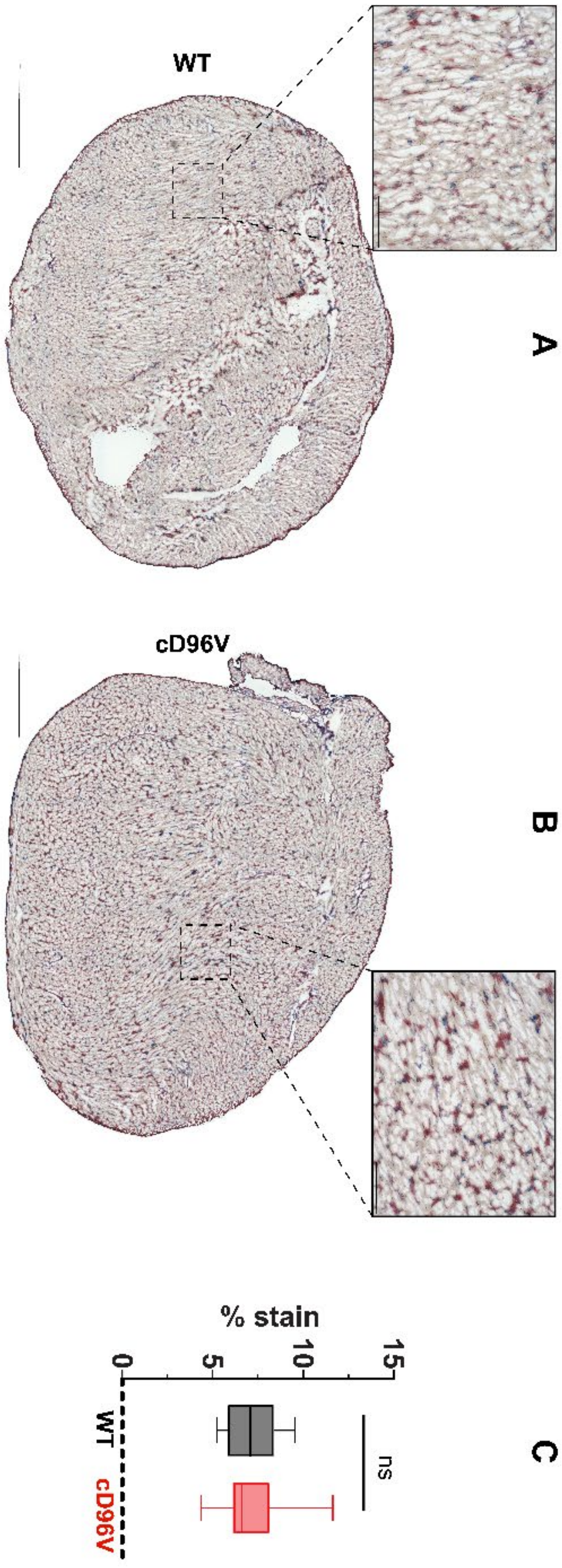
WT
cD96V
A
B
C
% stain
0
5
10
15
ns
WT
cD96V

Figure S18. Assessment of fibrosis in WT and cD96V myocardium. Representative images of Masson`s trichrome-stained heart sections of old (21-25 week old) **(A)** WT and **(B)** FLAG-tagged cD96V mice. Collagen is stained blue. Scale bars: 12.5 µm. **(C)** Summary data for % of tissue stained for collagen. For WT N = 3 (1 male, 2 females, 22 weeks old), for cD96V N = 3 (1 male, 2 females, 21 – 31 weeks old), 4 tissue slices per mouse for each group. ns – non-significant, $p > 0.05$ unpaired Student's t-test).

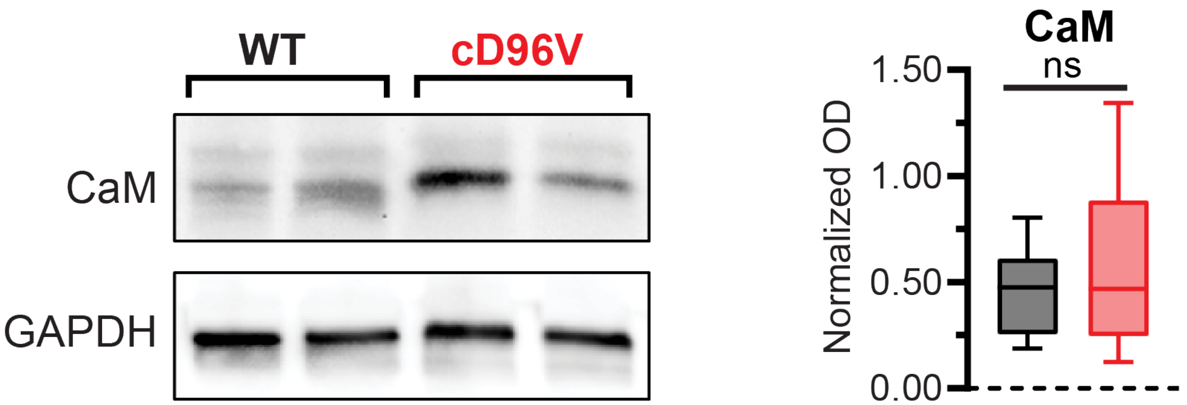

Figure S19. Protein levels of CaM in WT and cD96V myocardium. Representative Western immunoblots (left) and summary data (right) demonstrate similar total CaM expression levels in WT and FLAG-tagged cD96V hearts. OD – optical density. OD values of CaM blots were normalized to respective OD of GAPDH bots, N = 9 for WT (3 males, 6 females, 7 – 24 weeks old) and cD96V (3 males, 6 females, 7 – 23 weeks old), ns – non-significant ($p > 0.05$, unpaired Student's t-test).

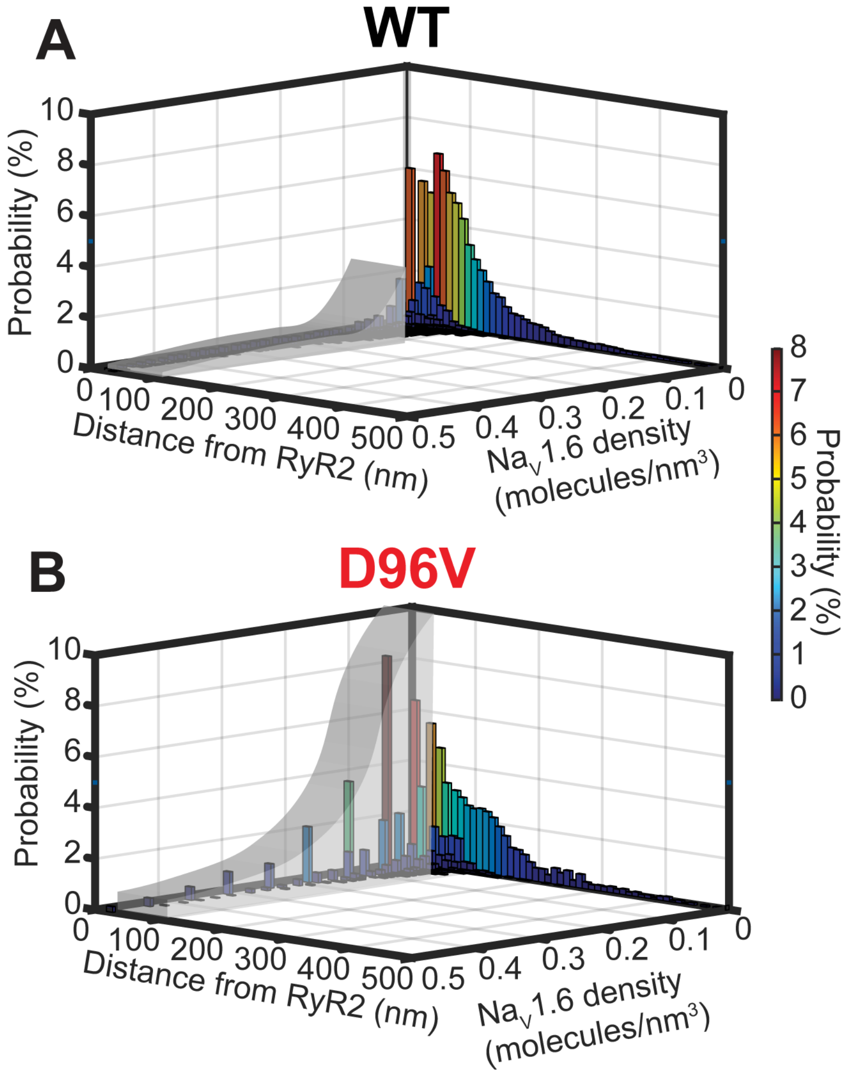

Figure S20. Density distributions of Na$_V$1.6 relative to proximity RyR2 in murine hearts. STORM-based relative localization analysis (STORM-RLA) analysis of Na$_V$1.6 cluster density versus Na$_V$1.6 distance from RyR2 in **(A)** WT and **(B)** FLAG-tagged cD96V hearts. Shaded region highlights differences between Na$_V$1.6 densities in relation to RyR2. n = 3 replicates, N = 3 for WT (1 male, 2 females, 22 weeks old) and for cD96V (1 male, 2 females, 21 – 31 weeks old).

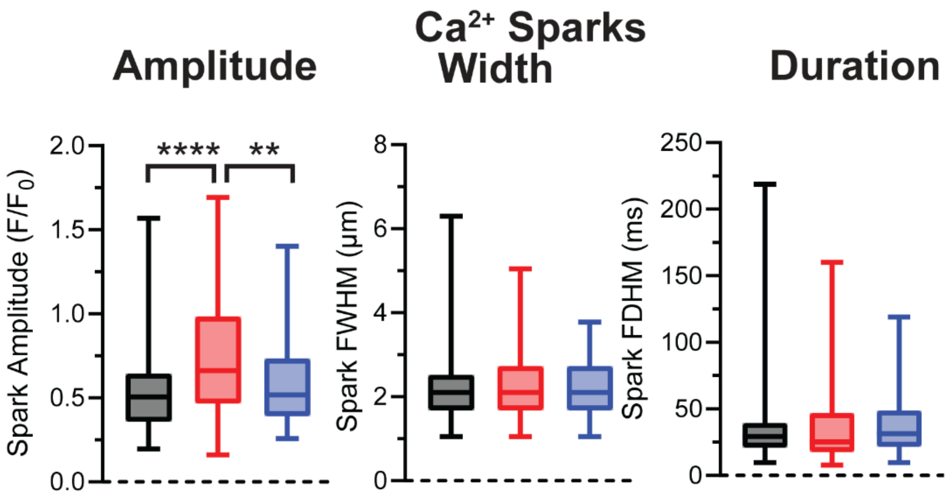

Figure S21. Ca^{2+} sparks characteristics. Ca^{2+} spark parameters from unrelated experiments: amplitudes (*left*), full width at half maximum (FWHM) (*middle*), and full duration at half maximum (FDHM) (*right*). ****$q < 0.0001$, **$q < 0.01$ Kruskal-Wallis test with original FDR method of Benjamini and Hochberg for multiple comparison. For WT n = 96, N = 13 (7 males, 6 females, 8 – 23 weeks old); FLAG-tagged cD96V n = 106, N = 10 (4 males, 6 females, 10 – 26 weeks old); and FLAG-tagged cD96V x cNav1.6KO n = 74, N = 8 (5 males, 3 females, 6 – 26 weeks old).

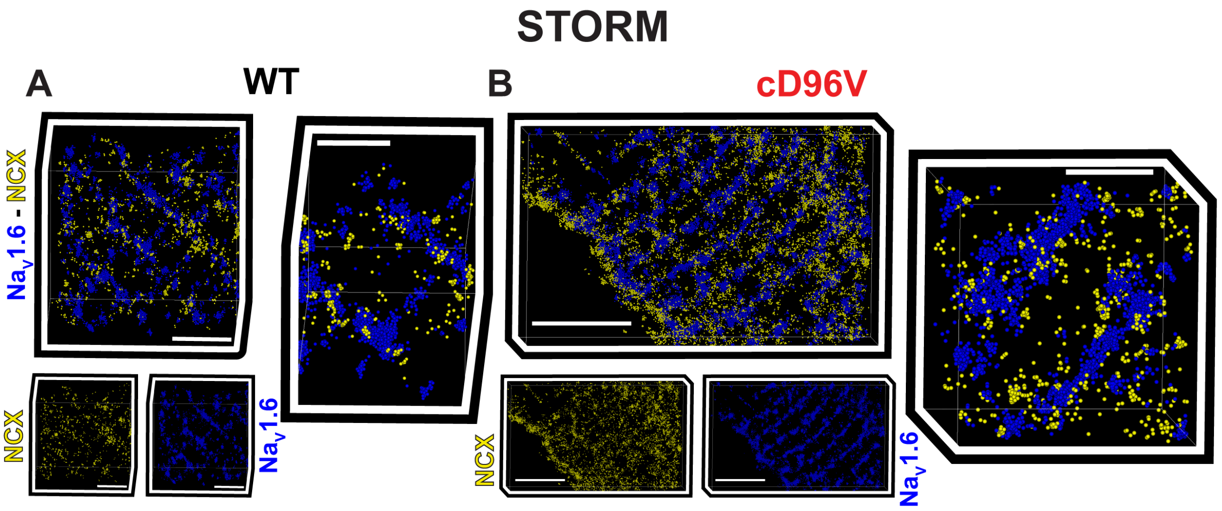

Figure S22. Close proximity (<100 nm) between Na$_V$1.6 and NCX in WT and cD96V hearts. Representative STORM images from **(A)** WT (a female, 22 weeks old) and **(B)** FLAG-tagged cD96V (a female 24 weeks old) hearts immunolabeled for NCX (yellow) and Na$_V$1.6 (blue). Scale bars: left 2 μm, right 1 μm in A and left 4 μm, right 1 μm in B.

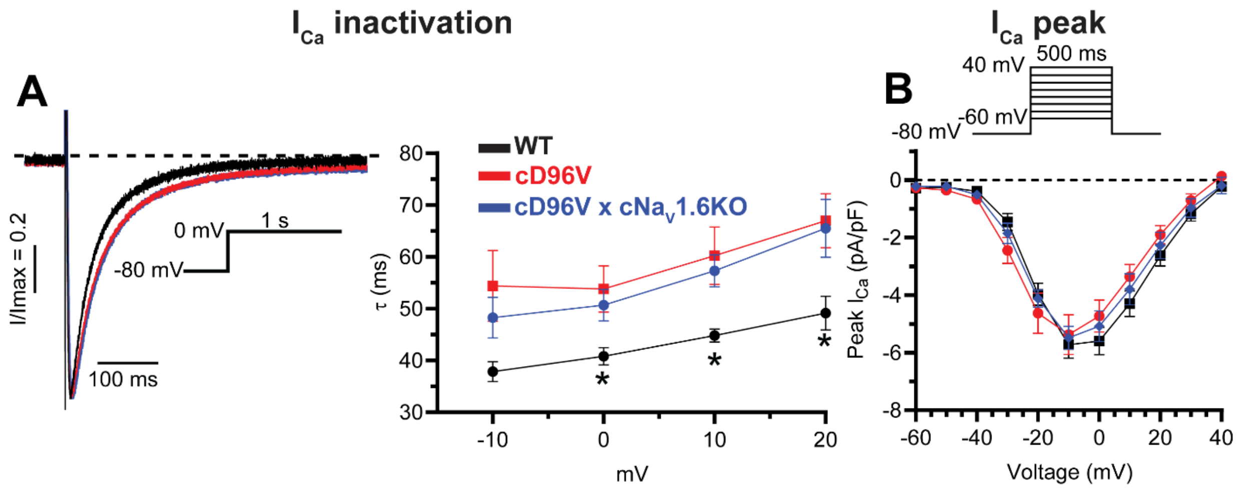

Figure S23. Cardiac-specific expression of D96V-CaM impairs I_{Ca} inactivation. A) Representative I_{Ca} traces (each trace normalized to its maximal I_{Ca}) and corresponding time constants of inactivation (τ). *$q < 0.05$ Kruskal-Wallis test with original FDR method of Benjamini and Hochberg for multiple comparison. **B)** Summary I-V curves of peak I_{Ca}. I_{Ca} was recorded in Na^+-free bath solution to eliminate I_{Na}, which was confirmed with the absence of currents at -40 mV. For WT n = 8, N = 4 (3 males, 1 female, 18 – 25 weeks old), FLAG-tagged cD96V n = 9, N = 5 (3 males, 2 females, 11 – 23 weeks old), FLAG-tagged cD96V x $cNa_V1.6$ n = 7, N=4 (2 males, 2 females, 12 – 24 weeks old).

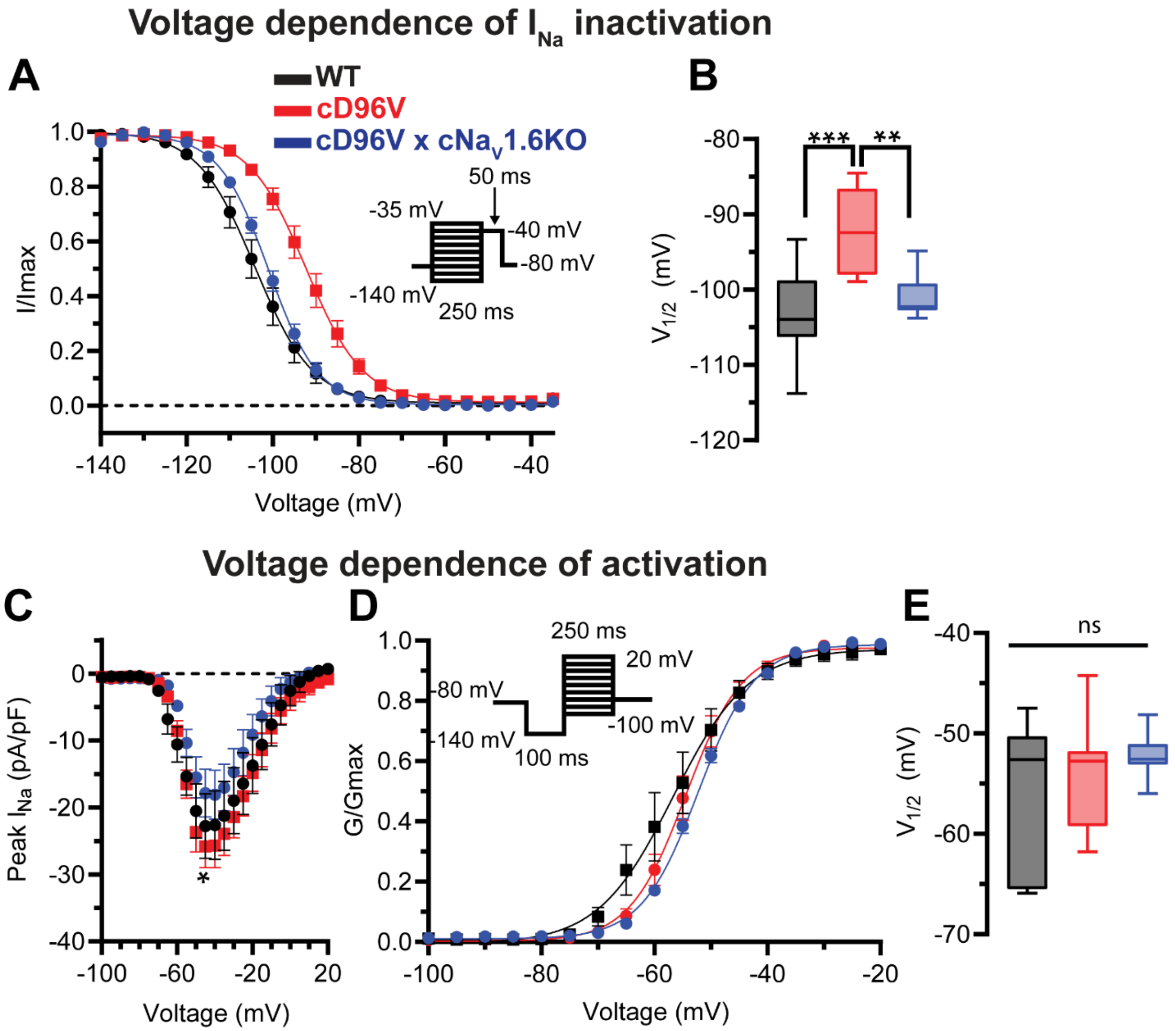

Figure S24. Cardiac-specific expression of D96V-CaM promotes Na$_V$1.6-mediated I_{Na} dysfunction. (A) Steady state inactivation curves and **(B)** corresponding $V_{1/2}$. For WT n = 11, N = 5 (2 males, 3 females, 6 – 14 weeks old); FLAG-tagged cD96V n = 12, N = 6 (4 males, 2 females, 10 – 25 weeks old); FLAG-tagged cD96V × cNa$_V$1.6 n = 10, N = 5 (2 males, 3 females, 7 – 25 weeks old). ***q < 0.001, **q < 0.01 with Kruskal-Wallis test with original FDR method of Benjamini and Hochberg for multiple comparison. **(C)** I-V and **(D)** G-V relationships with **(E)** corresponding $V_{1/2}$ of activation. For WT n = 9, N = 6 (2 males, 4 females, 6 – 23 weeks old); cD96V n = 14, N = 6 (3 males, 3 females, 10 – 25 weeks old); cD96V×cNa$_V$1.6 n = 12, N = 6 (4 females, 2 males, 7 – 25 weeks old). *q < 0.05 for peak I_{Na} density in cD96V comparing to cD96V×cNa$_V$1.6 at -45 mV and ns (not significant) with Kruskal-Wallis test with original FDR method of Benjamini and Hochberg for multiple comparison.

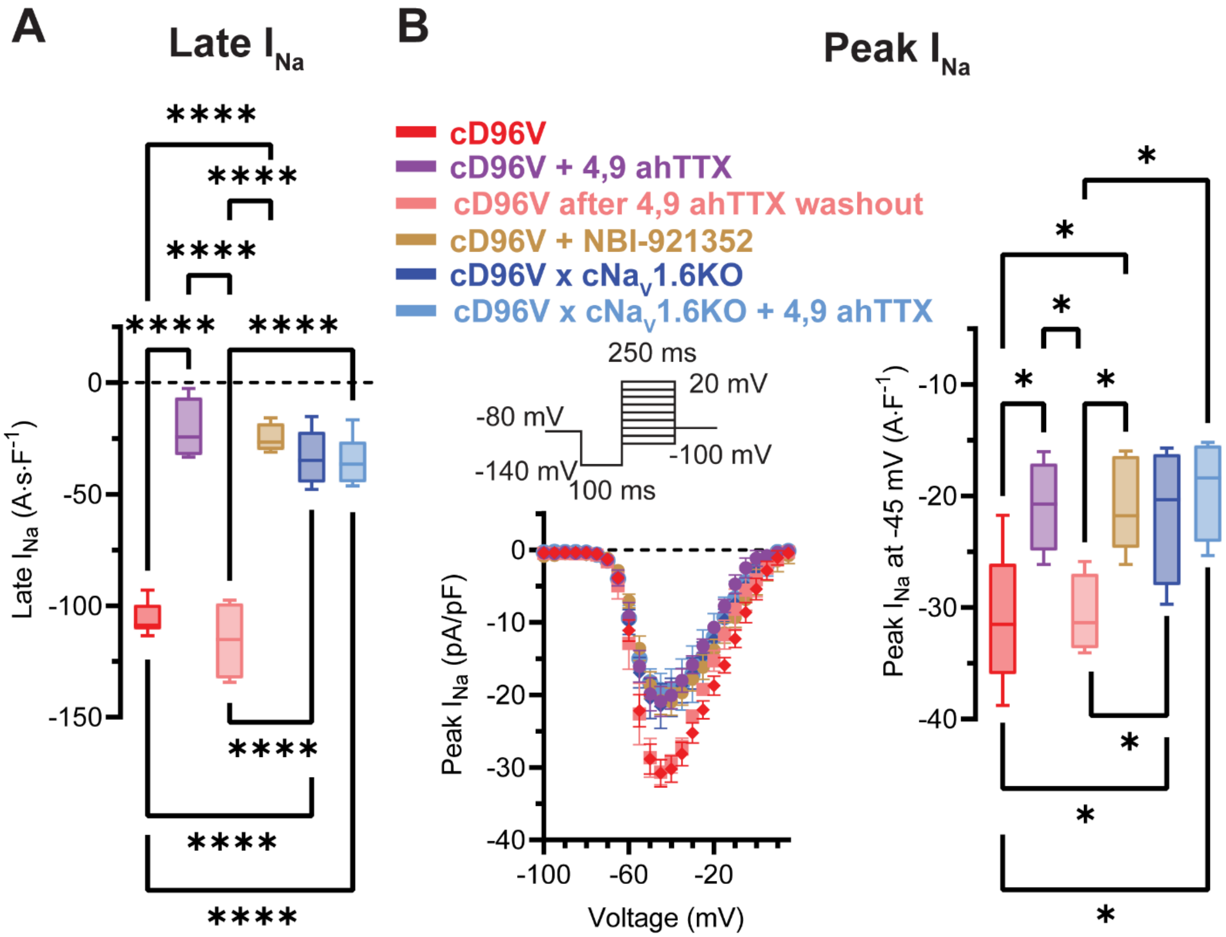

Figure S25. 4,9ahTTX and NBI-921352 ameliorate Na$_V$1.6-mediated late I$_{Na}$ in cD96V cardiomyocytes. Summary data of **(A)** late and **(B)** peak I$_{Na}$ attenuation in Flag-tagged cD96V myocytes by 4,9ahTTX (300 nM), NBI-921352 (1 μM) and cD96V x cNa$_V$1.6KO. **(B)** I-V relationships (mean ± SEM, *left*) and (*right*) peak I$_{Na}$ density at -45 mV. For late I$_{Na}$: cD96V n = 8, N = 4 (1 male, 3 females, 17 – 21 weeks old); cD96V + 4,9ah TTX and cD96V after washout n = 4 (paired experiments), N = 2 (2 females, 19 and 21 weeks old, respectively); cD96V + NBI-921352 n = 4, N = 2 (1 male, 1 female, 17 and 20 weeks old, respectively); cD96V × cNaV1.6KO and cD96V × cNaV1.6KO + 4,9ah TTX n = 5 (paired experiments), N = 2 (2 males, 16 weeks old). For peak I$_{Na}$: cD96V n = 9, N = 7 (4 males, 3 females, 17 – 20 weeks old); cD96V + 4,9ah TTX n = 4, N = 4 (3 males, 1 female, 19 - 20 weeks old); cD96V + NBI-921352 n = 5, N = 3 (1 male, 3 females, 17 - 20 weeks old); cD96V × cNaV1.6KO and cD96V × cNaV1.6KO + 4,9ah TTX n = 4 (paired experiments), N = 2 (2 males, 16 weeks old). **** $q < 0.0001$, *$q < 0.05$ by ordinary one way ANOVA test with original FDR method of Benjamini and Hochberg for multiple comparison.

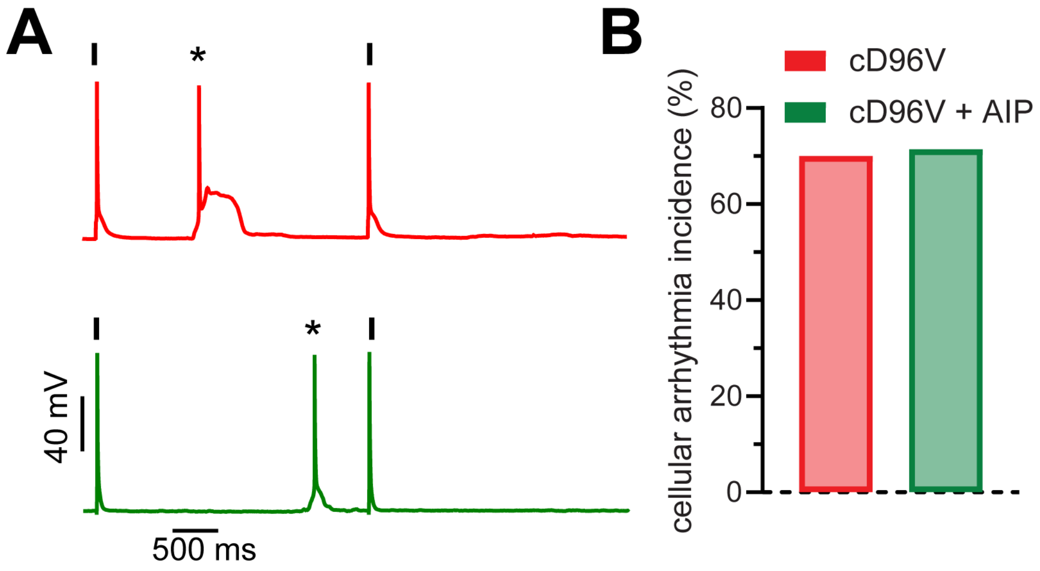

Figure S26. The effect of CaMKII inhibition on cellular arrhythmia in cD96V cardiomyocytes. (A) Representative action potential (AP) recordings from Flag-tagged cD96V cardiomyocytes in control conditions (red) and after 20 minutes pre-incubation with CaMKII inhibitor (myristoylated autocamtide-2-related inhibitory peptide, AIP; 10 µM; green). Current stimuli are marked by vertical bars, pacing frequency 0.3 Hz. Triggered activity marked with asterisks. **(B)** Cellular arrhythmia incidence, defined as the % of studied cells exhibiting delayed (DADs) and/or early afterdepolarizations (EADs) during AP recording. cD96V, control conditions (red, n = 10, N = 4, 3 males, 1 female, 9 – 23 weeks old). cD96V after 20 minutes pre-incubation with myristoylated AIP (10 µM, green, n = 7, N = 3, 3 males, 9 – 22 weeks old). $p > 0.05$ Fisher`s exact test.

Supplemental table 1. ITC parameters of CaM - Na_Vs binding

Na_VCTD-CaM	Kd (nM)	ΔH (kcal/mol)	ΔS (cal/mol·K)	n value	n
**0 Ca^{2+}**					
**Na_V1.5 CTD**					
WT-CaM	33.25±3.404	-16.00±0.5115	-19.40±1.689	1	4
D96V-CaM	35.83±1.639	-15.10±0.3391	-16.55±1.097	1	4
**Na_V1.6 CTD**					
WT-CaM	243.00±1.20	-18.50±2.400	-31.90±8.000	1	2
D96V-CaM	336.80±14.20*	-19.43±1.193	-35.50±3.998	1	4
**10 μM Ca^{2+}**					
**Na_V1.6 CTD**					
WT-CaM	132.3±11.02	-11.08±0.2450	-5.660±0.9499	1	3
D96V-CaM	239.7±27.20*	-15.74±0.3941	-22.45±1.521	1	3

Footnotes: mean ± SEM, for K_d of WT-CaM:hNa_V1.6 CTD vs. D96V-CaM:hNa_V1.6 CTD at 0 Ca^{2+} and 10 μM Ca^{2+} *$q < 0.05$ with one way ANOVA with Original FDR method of Benjamini and Hochberg for post hoc comparison.
